# Circular Quality of Polymers: Test-Based Evidence for Comparison of Bio-Based and Fossil-Based Polymers

**DOI:** 10.3390/polym17121629

**Published:** 2025-06-12

**Authors:** Ilija Sazdovski, Ferran Serra-Parareda, Marc Delgado-Aguilar, Leonidas Milios, Sahar Azarkamand, Alba Bala, Pere Fullana-i-Palmer

**Affiliations:** 1 UNESCO Chair in Life Cycle and Climate Change ESCI-UPF, Passeig Pujades 1, 08003 Barcelona, Spain; leonidas.milios@ec.europa.eu (L.M.); sahar.azarkamand@esci.upf.edu (S.A.); alba.bala@esci.upf.edu (A.B.); pere.fullana@esci.upf.edu (P.F.-i.-P.); 2 LEPAMAP-PRODIS Research Group, University of Girona, Maria Aurèlia Capmany, 61, 17003 Girona, Spain; ferri.espi@gmail.com (F.S.-P.); m.delgado@udg.edu (M.D.-A.)

**Keywords:** polymer properties, recycling quality, Qs/Qp factors, circularity, multiple recycling

## Abstract

The factual circularity of materials needs utilization of materials that keep their quality properties after going through recycling to minimize the inflow of virgin materials in the technosphere. Within the PEF methodology, and based on the economic model approach, the European Commission provides default parameters for the quality changes of the polymers after recycling, but solely for some fossil-based polymers. This study provides a test-based example for the calculation of technical substitutability for 2 fossil-based polymers (HDPE and PET) and 2 bio-based polymers (PLA and PHB) based on mechanical, processing, and optical properties. The results show that the economic substitutability method gives very different results compared to those obtained by considering real technical quality changes of the polymers in multiple-cycle recycling. HDPE proved to have superior circular properties compared to any other polymer under research. In addition, the recent practice of substituting fossil-based with bio-based polymers will need to be re-evaluated after additional research related to the quality change of bio-based polymers in recycling. The results showed the important limitations of the economic substitutability method and a strong need for a harmonized testing method for the calculation of quality degradation of all types of polymers in recycling.

## 1. Introduction

Numerous sectoral reports and scientific literature warn about the circularity gap of plastic materials entering the global market. Based on the Global Plastics Outlook report published in 2022, only 9% of the world’s plastics put on the market are recycled [1]. The same report warns that approximately 71% of plastics globally end up in landfills or finalize their life cycle as uncontrolled litter. The production of plastics has been soaring since its introduction on the market in the 1950s, and global production is rising every year. According to the latest data, the production of plastics in 2022 increased by 1.6 percent in one year, resulting in 400.3 million metric tons introduced to the market worldwide [2]. As a result of these non-circular and currently non-sustainable ways of treating plastic waste, the scientific literature claims that 12,000 million metric tons of plastic materials will be in landfills or the natural environment in 2050 [3]. This is a result of not economically viable circular paths, regardless of the recycling technologies, limiting the global adaptation of sustainable pathways of usage and treatment of plastic products [4].

The European Commission undertook a leading role in the high ambition coalition to end Plastic Pollution by the establishment of global rules to end plastic pollution [5]. The Circular Economy Action Plan [6] under the Green Deal represents the most comprehensive effort so far to limit the inflow of plastic waste into the environment. The European Strategy for Plastics in a Circular Economy, adopted in 2018, presents a set of strategic policy actions for turning the plastic challenges of today into a fully functional economy based on circularity principles [7]. As a result, the amount of post-consumer reprocessed plastics’ reuse/transfer into new products increased by 20% in 2022 compared to 2020 in EU27 + 3 [8]. Despite this fact, out of 24.5 million metric tons of plastic waste produced in the European Union, only 14% of plastic waste was recycled in 2020, providing 3.5 million metric tons of recyclate [9].

A specific limiting aspect of recycling plastics and, more concretely, thermoplastics is the loss of properties of plastics during mechanical recycling, the only well-established technological reprocessing of plastic waste material, mainly due to chain scission, hydrolysis, and thermal degradation during melt processing, while other aspects may also negatively influence this performance. This aspect might be crucial when deciding among the materials to be used and for calculating the circularity value creation principles established by the Ellen MacArthur Foundation [10]. The degradation of plastics during reprocessing limits the maximum recycling cycles that the material can stand and requires the incorporation of virgin material to fulfill the technical requirements, functionality, and overall quality for market acceptance, thus limiting the overall circularity of the plastics value chain [11].

Beyond waste collection difficulties, as reported in the literature, the subsequent recycling of plastics presents a challenge due to polymer cross-contamination, the presence of additives [12], non-polymer impurities, and polymer degradation [13]. Research developed by [14] studied the changes in the properties of polyethylene terephthalate (PET), polypropylene (PP), and polyethylene (PE) samples from household waste. They concluded that PET is suitable for closed-loop multiple recycling and, recycling of PE and PP resulted in difficulties in processability due to the non-homogeneity of the different packaging applications. Gaduan et al. [15] tested the material properties of high-density polyethylene (HDPE) in multiple recycling cycles (10, 20, and 50), adding different amounts of virgin HDPE at each recycling cycle, demonstrating the feasibility of blending 50% recycled content and reproducing milk bottles for the UK market. The effects of the multiple recycling cycles on the properties of HDPE were investigated in the study of Jiun et al. [16], who found that after five repetitive recycling cycles, there was a decrease in the physical and mechanical properties, such as density and tensile properties, including Young’s modulus and yield strength. The effects of the 10 repetitive closed-loop recyclings of PET bottles with different recycled content (from 10% to 100%) were a research subject of the study developed by Brouwer et al. [17]. The study found that the presence of contaminants in multiple recycling reaches asymptotic values and depends on the recycling content and recycling schemes. The case of 100% recycling content is an exception, and linear dependency of particle contamination is observed.

With the rise of awareness of the effect of fossil-based plastics on the environment, the industry promoted bio-based polymers as a substitute [18]. However, in many cases, blends of fossil-based plastics with bio-based plastics are produced to improve the properties of the bio-based materials [19,20,21,22,23,24,25,26]. These efforts represent a valuable contribution to the field of development of new materials; however, in the practical application of the currently existing waste management systems, they are non-recyclable due to a lack of a stream for waste separation [27,28,29]. Outside of a company’s closed-loop recycling system, these materials can be considered non-recoverable after one cycle.

An emerging concern in the recent scientific literature relates to the additives and intentionally added substances in the production and recycling of polymers. Additives serve to improve the mechanical, optical, and chemical properties of the polymers, as well as to improve durability and UV and temperature resistance. However, their impact on human health and the environment is not fully understood [30,31]. Exemplary, the use of additives in PET production and recycling is stated in the first edition of the “Cradle to Cradle” book from 2002, recommending production and recycling processes without adding functional additives [32].

### 1.1. The Ongoing Discussion About the Substitutability of Recycled Materials

Substitutability of recycled materials and the loss of quality properties is a discussed topic in the scientific literature. Rigamonti et al. [33] concluded that the involvement of the substitution ratio in LCAs is of immense importance to show the real environmental impacts of PET, HDPE, and other polymer materials in recycling, resulting in an increase of 15–20% of all impacts. The authors suggested the development of correct substitution ratios for the improvement of the LCA methodology. A similar conclusion related to the importance of the involvement of quality factors in the LCA is also highlighted in other scientific studies [11,34,35].

#### 1.1.1. The Maximum Number of Recycling Cycles

One possible method for the calculation of the quality degradation factor is the maximum number of recycling cycles that materials can stand, as presented in the work of Rigamonti et al. [33]. The authors applied the ISO/TR 14049 [36] and calculated the quality factor of paper in 5 recycling cycles and used the following formula 1−11+number of cycles to calculate the replacement potential of the secondary pulp based on quality degradation.

#### 1.1.2. The Economic Approach (Market-Based Mechanism)

Another calculation method for the quality degradation factor is the economic approach of comparing the price ratio between the primary and the secondary material at the point of substitution.

To create an environment for the promotion of sustainable products, in 2013, the European Commission launched the Single Market for Green Products initiative based on the Product Environmental Footprint (PEF) as a common method for measuring the environmental performance of products [37]. PEF is fully based on the Life Cycle Assessment standardized methodology [38], with the main improvement of setting specific rules for certain economic sectors (PEF Category Rules).

Influenced by the circularity paradigm, the European Commission introduced the Circular Footprint Formula for the end-of-life stage of materials, with default values for quality degradation of the materials in recycling [39], influencing the allocation of environmental burdens and credits to suppliers and users within the lifecycle of product alternatives. If recycled material performs better, there will be more demand for such material, and its manufacturer will benefit with more environmental credits. One of the values needed to solve the Circular Footprint Formula is the Qs/Qp ratio (the relative quality of the secondary material, Qs, with respect to the one of the virgin material, Qp). These Qs/Qp default values for plastic materials were determined according to a market-based mechanism and, therefore, they should be subject to market fluctuations; however, they have been static since their first introduction in 2018. Specifically, the default values for the quality of the ingoing and outgoing recycled material at the point of substitution in the mechanical recycling of PP, PET and HDPE are set at 0,9 and for LDPE is set at 0.75 [40]. These values influence the market of recycled materials and should be evidence-based. It is of the utmost importance to evaluate the differences among the different plastic materials, which may be competing for certain markets. This approach is reported to have setbacks due to market price variations or when prices are non-existent or inaccurate [41].

### 1.2. Technical Substitutability

The most frequently used method for calculation of the quality changes in recycling is the technical substitutability of the material at the point of substitution [42,43]. Indicators of technical substitutability are developed for different materials. Steinmann et al. [44] developed a material quality indicator based on the energy use of recycled products compared to their virgin counterparts, giving an example of steel products. Barbato et al. [45] developed a combined method for the substitutability of recycled glass based on technical quality (considering the impurities and color contamination) and market applicability. Huysveld et al. [46] also developed a combined method for quantifying the overall substitutability based on the technical and market substitutability and applied it in the case of mechanical and chemical plastic recycling waste. Roosen et al., [47] developed an operational framework for the quantification of the quality of recycling using three dimensions: virgin displacement potential, in-use stock lifetime, and environmental impacts. The dimension of virgin displacement potential can be considered as technical substitutability and can be applied to multiple different materials (aluminum, paper and cardboard, plastics, glass, etc).

#### 1.2.1. Overall Substitution Potential of Materials

Most of the concepts for quantifying technical substitutability are based on the framework for substitution presented in the work of Vadenbo et al. [48]. The authors presented a definition of overall substitution potential (based on the influence of four ratios) with the following equation:

(1) $$\gamma =U^{rec}\times \eta^{rec}\times \alpha^{rec:disp}\times \pi^{vir}$$

where:

*γ* is the overall substitution potential;
*U*^*rec*^ is the physical resource potential (percentage of the target material in a given waste stream);
*η*^*rec*^ is the resource recovery efficiency (collection, sorting, and pretreatment);
α^*rec:disp*^ is the substitutability of recycled plastic to match the functional properties of the virgin material;
*π*^*vir*^ is the market response (expected displacement of the virgin plastic by the recycled material).

The coefficient α^*rec:disp*^ can be considered as the technical substitutability and can be calculated based on the technical (mechanical, thermal, optical, processing, chemical) properties as a ratio between the value for the recycled material and the displaced one or, in the case of plastics, the value for the virgin ones.

#### 1.2.2. Applied Substitutability Based on Different Applications of Plastic Materials

A similar approach is undertaken in the study of Demets et al. [49]. The authors take into consideration several key mechanical properties (tensile properties, elastic modulus, and tensile strength) as well as the processing-related properties: Melt-Flow Index (MFI) and Intrinsic Viscosity (IV) from the published literature.

For each key material property of a specific application, a scoring function is drawn that can return the degree of deviation of the actual value (by the recycled plastic) from the acceptable range of values (determined by relevant virgin plastics), with a score between zero and one. For example, injection molding applications require high MFI values, whereas extrusion processes demand much lower values. Based on the corresponding scores for each type of property of recycled plastic, two recycling quality (RQ) ratios or Qs/Qp factors (the quality of the secondary material divided by the quality of the primary material, keeping in mind that sometimes the value of the quality is inversely proportional to the value of the property) are obtained: processing (${RQ}^{proc}$) and mechanical (${RQ}^{mech}$) factors.

${RQ}^{mech}$ is a weighted sum of the scores of the mechanical properties, in which the weighting factors are based on the future application of the recycled material. Ultimately, in their paper, $\alpha^{rec:disp}$ is determined by the minimum of ${RQ}^{mech}$ and ${RQ}^{proc}$.

(2) $$\alpha^{rec:disp}=min[{RQ}^{mech},\;{RQ}^{proc}]$$

where

${RQ}^{proc}=f\left(F^{vir},\;F^{rec}\right)\;$–function based on the relevant flow properties *(MFI, IV),* and

${RQ}^{mech}=w_{E}f\left(E^{vir},\;E^{rec}\right)+w_{\sigma }f\left({\sigma_{y}}^{vir},\;{\sigma_{y}}^{rec}\right)+w_{a_{c}}f\left({a_{c}}^{vir},{a_{c}}^{rec}\right)+w_{\varepsilon_{b}}f({\varepsilon_{b}}^{vir},{\varepsilon_{b}}^{rec})$

w—weighting factors;
E—elastic modulus;
$\sigma_{y}$—yield strength;
$a_{c}$—impact strength;
$\varepsilon_{b}$—strain at break.

The same approach using weighting factors is undertaken in the paper of Shulte et al. [50] and applied in a case study of Low-density Polyethylene (LDPE) involving mechanical, transmission, thermal, and aesthetic requirements of the material.

Changes in optical properties manifested by discoloration are usually a limiting factor for the usage of high recycling content [11]. Therefore, the substitution ability of the recycled material based on the changes in the optical properties is a prerequisite for the calculation of the quality changes of the polymers.

### 1.3. Aim and Objectives of This Study

The goal of our research is to check whether some default values given by the Product PEF methodology on Qs/Qp ratios for relevant plastics are in line with the degradation of physical properties that affect the material’s function. The scope of the research has some boundary conditions: (i) a sufficient number of plastic materials being tested, i.e., two fossil-based: PET and HDPE, and two bio-based alternatives: Polylactic acid, also called Polylactide (PLA), and Polyhydroxybutyrate (PHB); (ii) a closed-loop recycling based on the “cradle to cradle principles”, i.e., without adding additives in closed-loop recycling; and (iii) a complete series of tests of mechanical, optical, and processing properties, finding the maximal number of recycling cycles that a material can undertake in the extrusion molding process without losing its minimum processing properties.

The selection of the fossil-based materials is based on the availability of quality factors in the PEF methodology. In order to compare the test-based approach for the determination of the quality factors and derive conclusions, at least two fossil-based materials are needed. PET and HDPE are selected because they are considered the most recyclable polymer materials, primarily used in packaging applications [3].

The selection of bio-based materials is based on the biodegradability of the polymers, which excludes the bio-PET and bio-HDPE as possible alternatives due to the fact that they do not solve the environmental pollution from non-biodegradability. The most promising bio-based degradable materials serving a possible function in the packaging sector indicated in the scientific literature are PLA and PHB [51].

In addition to assessing the accuracy and relevance of the values of Qs/Qp ratios against the physical properties of the four types of recycled plastics mentioned above, the additional specific aims are: (i) proposing alternative values of Qs/Qp ratios adapted to the specific plastic materials; (ii) investigating the maximum number of mechanical recycling cycles that PET, HDPE, PLA, and PHB could withstand for their use in the same application; and (iii) Comparing the different performances between fossil-based and bio-based polymers in multiple recycling cycles.

## 2. Materials and Methods

### 2.1. Methodology Calculations

Since the optical properties of the recycled material are so important for being accepted downstream in the value chain, it is crucial to improve the accuracy of the most complete protocol found in the literature [49] by transforming Equation (2) by involving the changes of optical properties as follows:

(3) $$\alpha^{rec:disp}=min[{RQ}^{mech},\;{RQ}^{opt},\;{RQ}^{proc}]$$

where:

${RQ}^{mech}$—function based on changes of the mechanical properties [%];
${RQ}^{opt}$—function based on changes of the optical properties [%];
${RQ}^{proc}$—function based on changes of processing properties [%].

The weighting factors, as used in Demets et al. [49], are useful if the future application of the recycled material is known and the relative importance of the type of property can therefore be assumed. However, this parameter is usually unknown to the LCA practitioners during the development of their analysis. The obtained parameters for the quality changes of the three types of properties can be considered as general maximal values (i.e., with weighting factors equal to 1); therefore, the material properties are compared using the measured data of the quality changes of the materials in each consecutive cycle without using weighting factors. The parameter $\alpha^{rec:disp}$ calculated in this experiment is based on the comparison of the property changes between recycled and virgin material.

Finally, the maximum number of recycling cycles is also considered as an additional processing factor, and it is calculated based on Equation (4), a formula developed by Rigamonti et al. [33].

(4) $$RQ=1-\frac{1}{n_{rc}+1}$$

The formula was used as a maximal quality degradation factor because our goal was not to identify the technological substitutability of the material to serve the same function but to identify the maximum number of recycling cycles that were possible to process the material through injection molding in the lab.

### 2.2. Experimental Testing

All tested polymers were subject to multiple recycling loops, and test specimens followed a predefined testing protocol as explained below.

(1) Shredding and washing. Cleaning the flakes to minimize/eliminate impurities, and characterization.
(2) Drying. Reduction of the water content below the recommended limits for extrusion.
(3) Processing in a kinetic mixer to produce pellets and characterization.
  - Melt-flow index (based on ASDM D1238 [52]).
  - Density (based on ISO 1183-1—Method B “liquid pycnometer” [53]).
(4) Injection moulding. Production of specimens for materials characterization and proposed tests.
  - Charpy impact testing (based on ISO 179 [54]).
  - Izod testing (based on ASTM D256 [55]).
  - Tensile testing (based on ISO 527 [56]).
  - Bending testing (based on ISO 178 [57]).
(5) Optical properties. CIELAB coordinates (based on ASTM D2244 [58]).

After this series of testing activities, the loop restarted at point (i) by shredding the injection-molded specimens (without the cleaning step). Only PET specimens were preheated to minimize the moisture content before molding. A maximum number of 8 cycles or 7 mechanical recycling cycles was implemented (a lower number if the material proved to be unprocessable in the injection molding). After each cycle, 40 specimens were produced from every material after every cycle for mechanical and processing properties, and 5 specimens were produced for the optical testing. All details related to the dimensions of the specimens, testing and processing equipment, and processing parameters are presented in the Supplementary Materials, Section SA.

The quality ratios between the recycled materials and the virgin ones were calculated using the property quality value of the first recycling cycle. The tested properties of subsequence cycles in order to: (a) know how the materials fulfill the statements given by the plastic sector of multiple recycling; (b) calculate the indicator on the number of cycles by Rigamonti et al. [33]; and see how fast the quality of the recycled plastic degrades in multiple recycling.

## 3. Results and Discussion

For the calculation of the changes in the polymers’ mechanical properties, measured values are used to produce the trending line of the measured properties. Based on the trendline, the calculated values are used for the assessment of quality changes.

As an example, in the Supplementary Materials (Section SB), Figure SB1 shows the average and range of the measured values of Young’s modulus of HDPE at each cycle, the calculated trending line, and the theoretical values and their deviation. The graph shows that this material property does not change much for HDPE; however, the results vary up and down from the virgin cycle, while, theoretically, Young’s modulus is expected to increase in every recycling cycle. This behavior may be due to the sensitivity of the equipment and the uncertainty of the measurements, which, apparently, is higher than the actual changes of the property. Therefore, calculating the trendline and using it to extract calculated data for the property change helps to compensate for the inaccuracy of the measurement.

All results are obtained under the conditions described in the corresponding testing standards; however, the accuracy might be affected due to absolute measuring error. Having a set of multiple measured values of each property in each recycling cycle helps decrease uncertainties deriving from the accuracy of the measuring equipment or laboratory conditions.

For each property, the average values and the range of every set are used to build a trending line, which illustrates the variability of the results and provides theoretically calculated values. As an example of the variability, the Supplementary Materials (Section SB), Figure SB1 shows the average and range of the measured values of Young’s modulus for HDPE and their deviation at each cycle, the calculated trending line, and theoretical values. These theoretical values for the virgin material (0.949 GPa) and the first recycled one (0.959 GPa) are used in Table 1 to provide the Q factor (0.989).

The graph shows that this material property does not change much for HDPE; however, the results vary up and down from the virgin cycle, while theoretically, the Young’s modulus is expected to increase in every recycling cycle. This behavior may be due to the sensitivity of the equipment and the uncertainty of the measurements, which, apparently, is higher than the actual changes of the property. Therefore, calculating the trendline and using it to extract calculated data for the property change helps to compensate for the inaccuracy of the measurement.

### 3.1. Changes in the Mechanical Properties

The mechanical properties of plastic materials usually determine the feasibility of using plastics in one application or another, although properties such as optical or thermal are also relevant and determining for specific applications. As introduced before, mechanical recycling usually leads to property reduction (e.g., strength, rigidity) due to successive melt compounding stages. This usually results in downcycling rather than recycling, tackling applications with lower technical requirements. Among mechanical properties, the most relevant and representative usually arise from tensile, flexural, and impact testing, resulting in tensile strength and Young’s modulus, flexural strength and modulus, and impact strength. The mechanical recycling process, usually during extrusion, results in chain scission and chain branching due to the submission of the melt polymer to high shear forces, as well as enhanced crystallization during molding and solidification [59,60].

**Figure 1:**
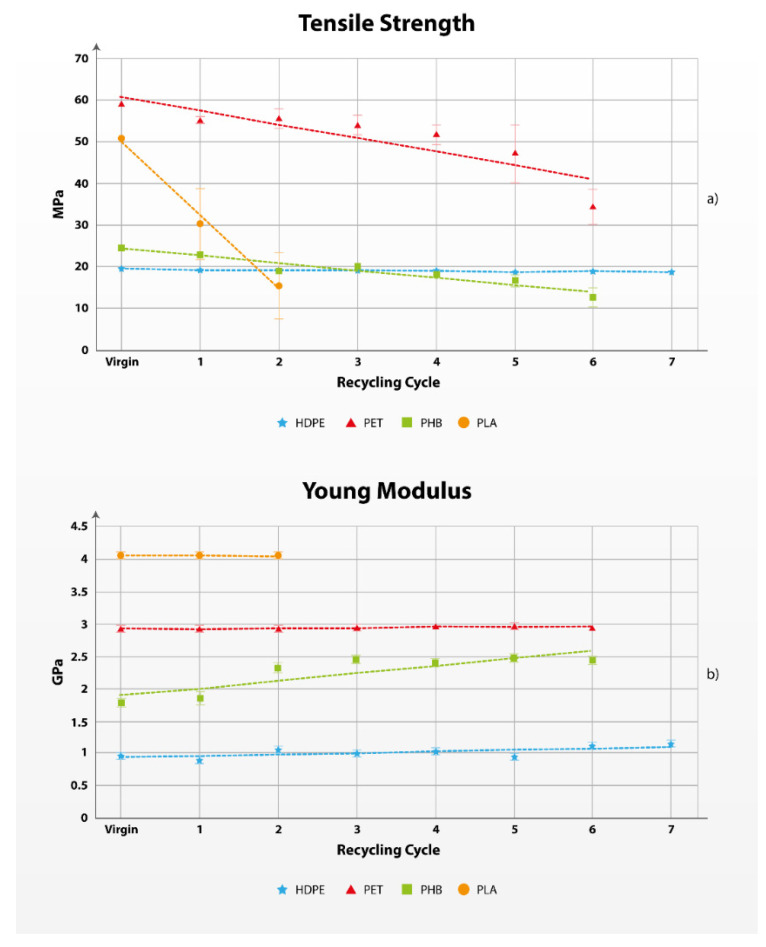
Evolution of the tensile strength (**a**) and Young’s modulus (**b**) of HDPE, PET, PHB, and PLA with mechanical recycling cycles.

**Figure 2:**
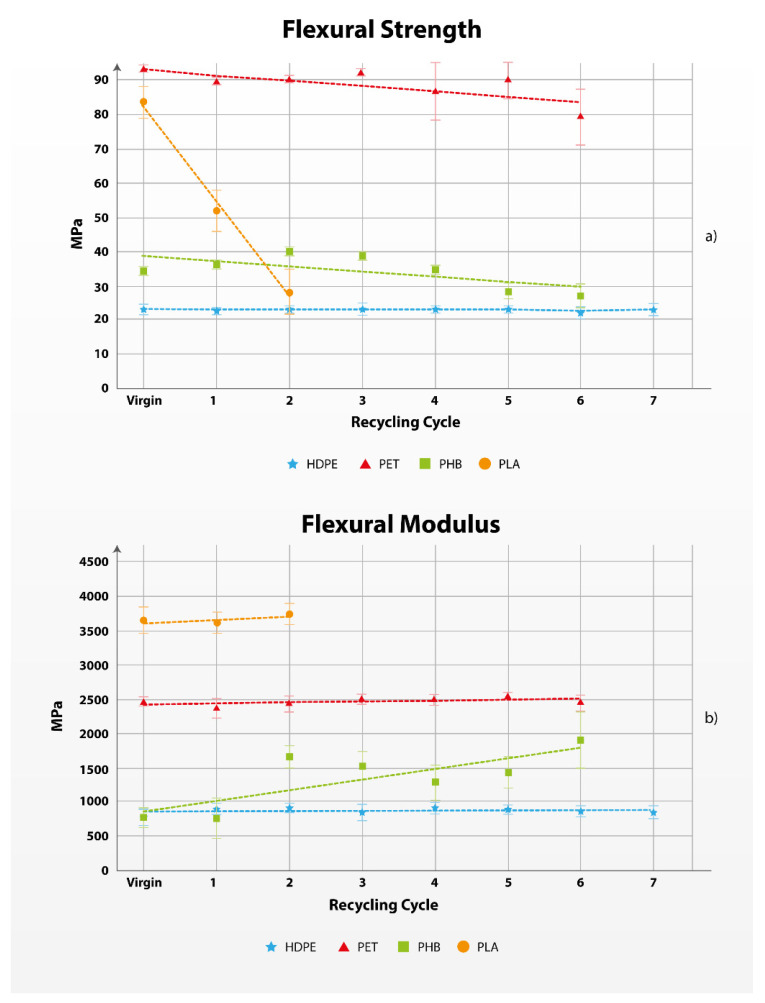
Evolution of the flexural strength (**a**) and flexural modulus (**b**) of the polymers in multiple recycling cycles.

The results presented in Figure 1 and Figure 2 revealed that HDPE exhibited the best performance during mechanical recycling, as the tensile and flexural properties remained constant regardless of the number of recycling cycles. This good performance during recycling can be attributed to the semicrystalline structure of HDPE, allowing the crystalline structures of HDPE to remain almost intact; the high thermal stability, with degradation temperatures approximately 300 °C, and minimal chain scission due to its linear structure so that, although some chains may be shortened, others remain intact [61,62].

Contrarily, still in the domain of oil-based and non-biodegradable polymers, PET experienced a reduction of 41.64% of the tensile strength after six recycling cycles. This can be attributed to its susceptibility to hydrolysis, thermal degradation, and oxidative breakdown, and chain scission [63,64,65].

These mechanisms lead to a reduction in molecular weight, intrinsic viscosity, and crystallinity, all of which contribute to its weakening compared to HDPE. Additionally, PET is more sensitive to the formation of degradation by-products, such as acetaldehyde and carboxyl end-groups, further compromising its strength and durability after multiple recycling cycles.

Referring to bio-based and biodegradable polymers, these are PLA and PHB; the former was only able to stand for two recycling cycles, evidencing serious processability problems. Exemplarily, the standard specimens were broken during the ejection process from the mold, making their testing unfeasible. This can be attributed to PLA hydrolytic degradation during recycling, resulting in a significant decrease in the molecular weight [66].

The latter exhibited better processability during recycling, although the tensile strength also experienced a reduction with the successive cycles. PHB exhibits a melting point between 160 and 170 °C, while degradation temperature is usually reported approximately 210 °C, leading to processing temperatures excessively close to its initial degradation [67,68]. In addition, the polymer experiences random chain scission during mechanical recycling, resulting in a reduction of the molecular weight and, thus, having a direct impact on the mechanical performance of the material. Contrarily, Young’s modulus experienced a slight increase, mainly attributed to the increase in the brittleness of the material due to enhanced crystallinity and, thus, promoting the generation of defects and voids, but also changing the polymer chain orientations [69,70].

#### 3.1.1. Tensile Strength and Young’s Modulus

Tensile properties are often related to the overall quality of the materials [71]. Some tensile properties are tensile strength, Young’s modulus, and strain at maximum strength and at break. Figure 1 provides the evolution of tensile strength, Young’s modulus, and strain at break of the selected polymer matrices as they are subjected to mechanical recycling. When plastic materials are under tensile stress, the material usually elongates and deforms. There are two types of deformation: (i) elastic deformation, when the material has the ability to return to its original dimensions; and (ii) plastic deformation, when the material does not return to its original form. A specific indicator is the maximum deformation of the material before fracture. An additional important property in tensile testing is the Young’s modulus. The Young’s modulus presents the stiffness of the material; the greater the modulus, the smaller the elastic strain [72].

In Table SC1 of the Supplementary Materials, we can observe the deformation at break of the materials in tensile testing. HDPE did not break in elongation of 100 mm in all recycling cycles, and PET did solely during testing of the virgin material. All other specimens are fractured in a certain elongation, and the elongation at break is shorter as the recycling cycles increase.

The most significant calculated quality changes based on the tensile properties change can be observed within PLA results, specifically to tensile strength and maximum deformation, resulting in a quality ratio of 0.631 when the virgin and recycled properties are compared (as the minimum value among the three properties: 0.648; 0.631; and 0.999). The same property changes can be followed by the comparison of the maximum deformation of the material before fracture. The changes in the Young’s modulus of all materials remain relatively stable, aside from PHB, where slight changes in the quality are observed. However, the Young’s modulus is not a limiting mechanical property index in multiple recycling of the polymeric materials under review. Detailed measured data from the tensile test are presented in Section SC of the Supplementary Materials.

#### 3.1.2. Flexural Strength and Flexural Modulus

The flexural properties of the materials are similar to the tensile properties, but they measure different aspects of the material. The flexural properties are manifested by measuring the flexural strength, flexural modulus, and maximal deformation. Flexural strength, or bending strength, is defined as the ability of the material to oppose deformation under load [72,73].

Figure 2 and Table 2 show the results from the flexural tests, and the steepness of the trendline shows that the flexural strength of PLA changes fastest in multiple recycling, while the flexural modulus changes fastest with PHB. The deformation at break is also measured, and the data is available in Section SD in the Supplementary Materials, but without a significant contribution to the results from the qualitative changes of the material.

The test measurement results show insignificant change in the flexural properties of the fossil-based materials, while bio-based materials show substantial change, especially the flexural strength and maximal deformation of PLA, and the flexural modulus of PHB. Detailed measured data from the tensile test are presented in Section SD of the Supplementary Materials section.

#### 3.1.3. Impact Tests

Impact properties are important mechanical properties of the polymers. Impact tests define the performance of the material under immediate and fast stress. During testing, the impact resistance is calculated based on the energy absorbed by the material specimen during the impact process [74].

Usual tests performed to calculate the impact properties of the polymers are unnotched and notched impact tests. The difference between the two is the preparation of the material specimens, where the weakened (notched) part of the specimens is on the opposite side of the impact. In addition, the measurement of the crack propagation resistance is usually performed using the IZOT test with unnotched specimens. All measured data in Table 3 are obtained using the pendulum test.

HDPE showed stable performance in the impact tests, without breaking the specimens and without previous weakening by creating a notch, therefore, for the Charpy unnotched test and IZOD, the measurements are non-existing. The kinetic energy of the pendulum was not sufficient to damage the specimens. Similar properties can be observed in PHB materials for the Charpy unnotched test in the virgin and 1st recycling cycle. PET specimens behaved similarly in the virgin testing of Charpy Unnotched and the virgin and 1st recycling cycle of IZOD testing.

**Figure 3:**
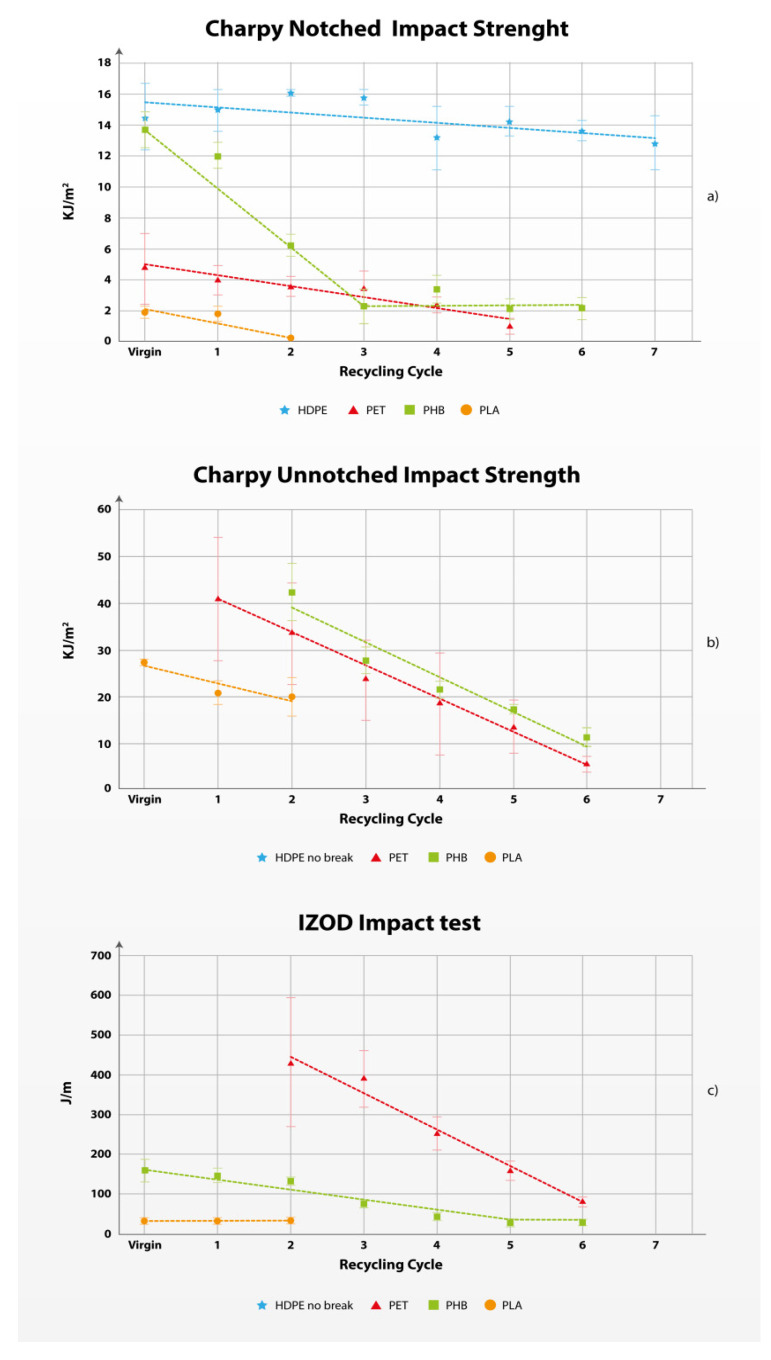
Evolution of the Impact properties: Charpy notched impact strength (**a**), Charpy unnotched impact strength (**b**) and IZOD impact test (**c**) of the polymers in multiple recycling cycles.

Besides the fact that the kinetic energy of the pendulum was not sufficient to break the specimens, from Figure 3, the material quality degradations are observed, therefore, the substitutability was not calculated as a ratio between the measurement of the 1st cycle and the virgin, but with the first cycle possible. Therefore, for the Charpy unnotched test, for PET we measured the ratio between the 2nd and the 1st recycling cycle, and for the IZOT test, the ratio between the 3rd and the 2nd recycling cycle. A similar calculation method is applied for the PHB Charpy unnotched test.

The testing of specimens in the 6th cycle of PET was impossible due to the sensitivity of the pendulum measuring equipment. The quality of the material decreased to the extent that the kinetic energy lost for breaking the specimen was so low that it could not be registered by the pendulum.

During impact testing, one manifestation that is specific solely to this testing property can be observed. The results obtained for the PHB notched test between the 3rd and 6th recycling cycles, and the IZOD between the 5th and the 6th cycles, as well as the PLA IZOD test, are so similar that a statistical analysis is necessary to be conducted. Anova single-factor statistical formula provides a comparison of variances between the tested results to highlight the differences between the measured results. The analysis is presented in the Supplementary Materials section, Section SF. The results show non-statistical variance in the mentioned groups of measured results. Simply, the same result is measured, and the variations occur due to small relative errors in the measurements. For all these groups of measurements, the average number for the calculation of the quality factor is used. This can be observed as a horizontal change in the trendline of this measurement in Figure 3. For applications where this IZOD is crucial, the maximal recycling cycles for PHB can be considered as 5 cycles or 3 cycles related to charpy notched and quality factor appropriately calculated.

The calculated quality change of the impact properties is the highest with HDPE (0.98) in the Charpy notched test and the lowest in PHB (0.729). The slope of the quality change in Figure 3 shows that, besides the Charpy notched test, PET shows a similar change in the quality degradation as the biobased polymers. The lowest calculated values of the quality degradation for PLA are observed in the Charpy unnotched test (0.76), and for PET in the IZOD testing properties (0.79).

#### 3.1.4. Density Test

The density of polymers follows the definition in classical mechanics as the mass of material per unit volume and is a convenient measure of the degree of crystallinity [69]. Density is a property that can be attributed to changes in other mechanical properties of the polymers and is especially useful for comparison of the property changes in polymer blends [72]. For the determination of the density of the polymers, a pycnometer test based on the ISO 1183-1–Method B [53] was performed. From the data obtained, we can see that all results are in the ranges defined in the literature, where HDPE has the lowest value (appx. 0.9 g/mL) and PET has the highest value (appx. 1.3 g/mL) [75].

**Figure 4:**
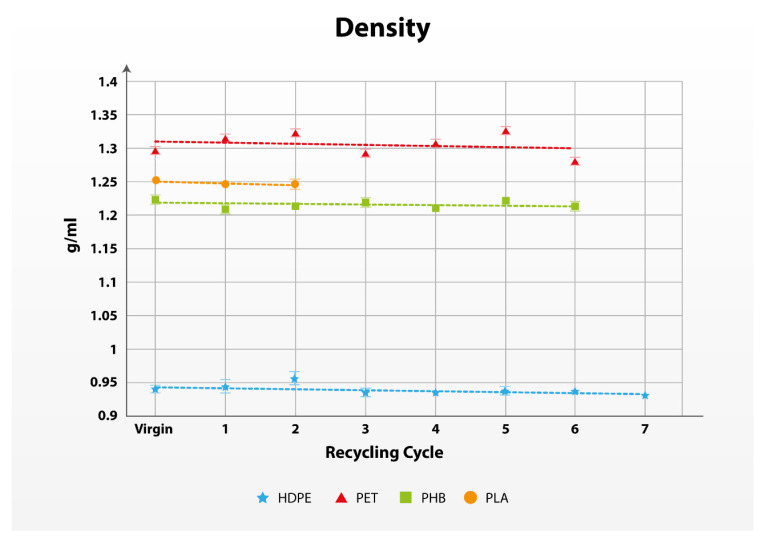
Evolution of the density of the polymers in multiple recycling cycles.

Almost a minimal slope in the trendline in Figure 4 can be observed, showing minimal changes in the density of all observed polymers. The quality degradation factor calculated in Table 4 shows that the density is the most stable mechanical property in all recycling cycles. The calculated quality degradation between the virgin material and the 1st recycling cycle is 0.99 in all materials. Only the comparison between the quality of the 7th recycling cycle and the virgin HDPE drops below 0.99.

### 3.2. Changes in the Processing Properties

#### 3.2.1. Melt Flow Index (MFI)

The MFI is one of the most important measurements for assessing the shear flow properties of melted plastics. The flow of melted plastics has the highest importance in the processing of plastics. The MFI is measured based on the rate of extrusion and its dependence on the temperature and weight [67]. During the experiment, HDPE was tested with a standardized mass of 2.16 kg and a standardized melting temperature of 210 °C. However, for all other materials, the standardized mass was impossible to measure with the equipment due to the fast deterioration of the material. In Supplementary Materials, Section SG, Photo SG1., a measuring problem of PHB is presented with a standardized weight observed in recycling Cycle 2. To overcome this measurement problem, the weight of the piston was decreased so that comparable results between the cycles can be observed in all recycling cycles.

**Figure 5:**
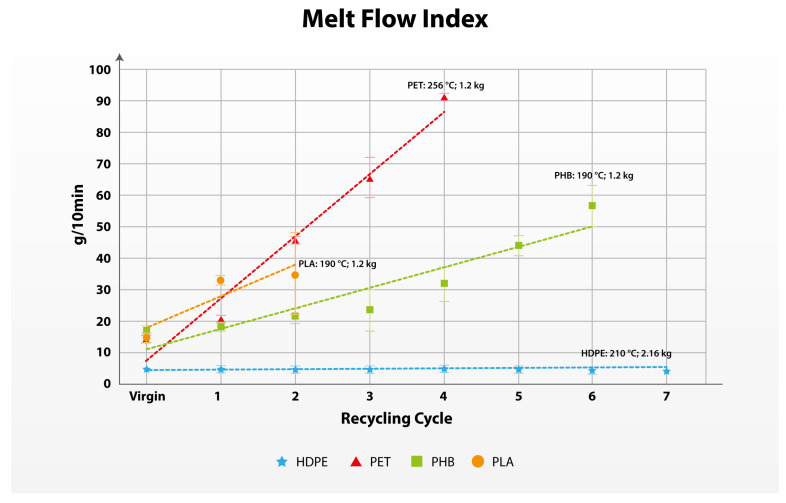
Evolution of the Melt Flow Index of the polymers in multiple recycling cycles.

As seen from the sharpness of the trendline in Figure 5, the MFI is one of the limiting properties of the tested polymer materials. Even with the decreased weight, PET samples were impossible to measure in Cycle 5 and Cycle 6. As can be seen from Table 5 and Figure 5, some cycles show slightly bigger standard deviations in the measurement, especially in the later recycling cycles, starting from Cycle 3 or Cycle 2 related to PLA.

Additionally, a slight discrepancy in the linearity of the changes in the material properties and solely between the Virgin and Cycle 1 for PET and PHB is observed. The difference between the calculated value based on the trendline and the measured data shows a slightly bigger discrepancy, and the data of the quality changes in a range (0.282–0.675 for PET and 0.644–0.928 for PHB) is presented where the lower figure represents the calculated value, and the upper figure represents the measured value. These measuring errors are stated in the scientific literature and are possibly due to slip at the barrel wall and end effects, but this method is proven to be very useful in assessing the effects of processing polymers [73]. From the measured data, the most stable material is HDPE based on these flow processing properties.

#### 3.2.2. Maximum Recycling Cycles

Maximum recycling cycles are not calculated based on the requirements of the material properties for a future application, but rather the maximal recycling possible for processing the material through injection molding. The quality factor is calculated based on the formula developed by Rigamonti et al. [33] and presented in Equation (4) in the Methods section.

HDPE was processed without any problems for all seven recycling cycles. The initial virgin material for conducting the experiment was sufficient for a minimum of seven cycles because of the separation of material due to the production of specimens (appx. 1 kg per cycle).

PHB started with the processing problems in the 5th cycle and especially in the 6th recycling cycle. The specimens were broken and clogged the exit point of the material from the moulder, because the specimens were too rigid to be separated from the mould. Also, some losses of material occurred, and, although solely several specimens for the 7th recycling cycle are processed, the specimens did not pass the satisfactory qualification. All processing problems of PHB are documented and presented in Section SH of the Supplementary Materials, Picture SH1.

PET material, similarly to PHB, started with processing problems in the 5th cycle and, in the 6th recycling cycle, the material was too rigid for successful processing. The specimens were usually broken by the pressure of the extraction pistons from the mold. All processing problems of PET are documented and presented in Section SG of the Supplementary Materials section, Picture SH2.

PLA showed slight processing problems since the 1st recycling cycle, such as variation of the color of the specimens and the creation of air bubbles inside the specimens. In the 2nd recycling cycle, the material was practically impossible to process due to big losses. Additionally, the material was too difficult to process due to its rigidity. The specimens were broken inside the mould, the material frequently clogged the exit point of the moulder, and the extraction pistons broke the specimens. More importantly, the material clogged the moulder due to crystallization. All processing problems of PLA are documented and presented in Section SH of the Supplementary Materials section, Picture SH3.

Calculated values of the quality factors based on the maximal recycling cycles are presented in Table 6.

### 3.3. Changes in the Optical Properties

Changes in the optical properties represent the visual quality of the recycled polymers suitable for the production of a subsequent product. Changes in the optical properties do not interlink with the changes in the mechanical and processing material properties. There are known examples in the industry where the manufacturers disregard the optical properties of the polymers in order to maximize the amount of the recycling content without compromising the production possibilities of the final product (Picture SI1. in the Supplementary Materials, Section SI).

Optical properties as well as the visual appearance are determined through the changes of color, the nature of its surface, and its light transmission properties [75]. For the measurement of color changes, CIELAB is one of the most used methods by industries including paints, plastics, automotive, textiles, packaging, food, and cosmetics [76,77]. The color changes in the polymers in recycling express the changes in three categories (coordinates): (i) L*—lightness; (ii) a*—level of redness or greenness; and (iii) b*—level of yellowness or blueness. ∆E is a standard measurement—created by the International Commission on Illumination, that quantifies the difference between two colors, in our case, the difference between the specimens from two consecutive recycling cycles.

**Figure 6:**
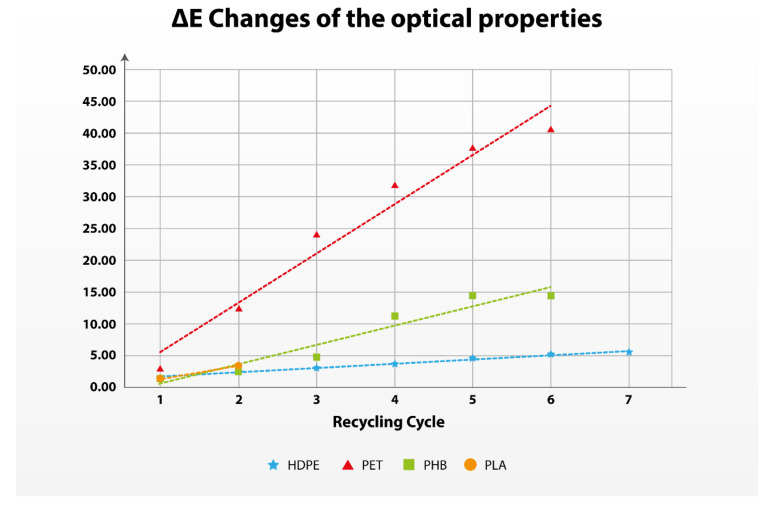
Evolution of the optical properties of the polymers.

In Figure 6, the changes in the optical properties of the polymers under research are observed, and as presented, the optical properties are one of the limiting properties of the polymers under research, especially the fossil-based polymers.

As presented in Table 7, the calculated quality changes in the optical properties are the lowest for the fossil-based polymers (PET and HDPE). This is probably a result of avoiding stabilizers in recycling that affect especially PET due to its high melting temperature. In Supplementary Materials, Section SI, Picture SI2., an obvious difference between the optical changes in PET is presented between the Virgin Material and the 1st recycling cycle.

### 3.4. Summarized Calculation of the Quality Degradation of the Polymers

Table 8 presents the summarized results of the quality degradation of the polymers under research, organized by type of property (mechanical, processing, and optical). Due to the type of recycling, the “cradle-to-cradle” method, and avoiding any thermal stabilizers during recycling, the optical properties showed to have limiting values, especially for PET. However, for the producers, it is easy to overcome these limitations because this property does not affect the production of the polymer product. It only affects the visual attractiveness to the buyers.

The optical properties proved to be a limiting factor for reprocessing the polymer materials under review. The biggest difference between the changes in the optical quality properties is noticeable with HDPE from a minimum quality factor of 0.98 in mechanical properties to 0.671 in changes in quality in optical properties. HDPE keeps the material properties with only slight changes, making it a suitable material for “cradle-to-cradle” recycling, but optical properties are the limiting factor. Optical properties and not decisive in the recycling possibility of the material, they only serve to keep the visual quality of the future application, therefore they do not limit the material to be recycled in multiple recycling cycles.

The mechanical properties are crucial for the future production of the subsequent products, and HDPE has almost perfect circular properties. The impact tests were limiting for the PET, decreasing the quality factor below 0.9. However, for biobased materials, especially PLA, the quality changes in the tensile and flexural properties may strongly limit the recycling content in the production of subsequent polymer products.

The low processing properties of PLA may also result in serious problems in the reprocessing of the recycling material. Both MFI and the maximum recycling cycles of PLA limited the substitution factor to 0.444. The substitution factor, due to the processing property changes of PET may not exceed 0.676. This limits the future reprocessing of PET materials in the “cradle-to-cradle” model, keeping in mind that PET is the polymer with the highest demand for recycled material in the market.

## 4. Conclusions

Recycling our waste and making a new resource out of it is one of the options given by the Circular Economy framework. Due to their high visibility, massive production, and difficulty with other management alternatives, plastics are a material with a great need for recycling. Recyclability is based on different factors, such as the difficulty of the collection stage, the low price of the virgin material, a proper design for recycling, and the quality of the secondary resource obtained in comparison to the virgin alternative (Qs/Qp ratio).

The definition of the relative quality of the secondary material and the virgin one influences the results of LCA studies when comparing material alternatives to fill in a certain market niche. Moreover, since the European Commission Product Environmental Footprint Circular Formula uses default values for the Qs/Qp ratios of materials based on old economic data and does not discriminate per type of plastic, it is of the utmost importance to use values that are related to the functions of the material through its properties. This paper presents a method for the establishment of the quality degradation of polymers based on the technical substitutability of the material. The conclusions of this study can be summarized in five separate outcomes:

(i) There is a need for updating the Qs/Qp factors for fossil-based polymer materials in the PEF methodology developed by the European Commission. The first outcome of the study is the lower values obtained for the Qs/Qp ratios by testing physical properties in comparison to the ones used by the PEF methodology and calculated through the market approach. For PET and HDPE, the default values are 0.9 for the plastic material going through mechanical recycling, but the physical property tests show a different reality, with a Q factor of 0.328 for PET and 0.671 for HDPE. As a consequence, plastic materials seem to be favored by the current PEF default values. Further research into other materials is needed to confirm this statement. With PET being the most commonly recycled plastic, it is relevant to say that the PEF default values (which are the same for PET and HDPE) benefit PET compared to the second most recycled, HDPE, as the quality changes in recycling are relevant between these two fossil-based polymers.
(ii) The second outcome of this study is related to the latest trend for the substitution of fossil-based polymers with bio-based ones. Currently, there is no recycling stream, and practical recyclability of bio-based polymers is non-existent. Moreover, even if the market for the recycling of bio-based polymers is created, the material properties decline so fast in consecutive recycling cycles that dilution with a large amount of virgin material or the massive use of additives will be needed to keep satisfactory properties, especially for the tested case of PLA. We cannot conclude that fossil-based plastics perform better than bio-based plastics because of the behavior of PET in multiple recycling. However, the melting-point temperature of PET is much higher than the other tested materials. Additional research with more bio-based and fossil-based materials in different testing conditions will have to be conducted for a unified conclusion.
(iii) The third outcome of this study is related to the need for the development of Qs/Qp factors for a wide range of bio-based polymers in the PEF methodological guidance.
  For the bio-based polymers, there are no PEF default values developed, and it is a question of how the practitioners can use the Circular Footprint Formula for them. The results of our study may support the development of Qs/Qp factors for PHB and PLA. Our first results indicate that PHB behaves approximately 20% better than PLA.
(iv) The fourth outcome of this study is related to the optical properties of the polymer materials in the multiple recycling process. The changes in the optical properties proved to be a limiting property in multiple recycling, even though they are not crucial in the functional ability of the material for the subsequent application. The results clearly show that to fulfill the requirements of the “cradle-to-cradle” recycling model, the producers of polymer products will have to change their perspective on the visual aspects of the products to maximize the circularity of the used materials. Thus far, the industry has supported “visually flawless” products as an identity for the quality of the products, but this non-circular practice will need to be changed and might even provide a business model for the producers. A similar visual perspective of the customers was changed, for example, for the paper-based industry when there was a simple visual identity of the paper using high recycling content.
(v) There is a strong need to overcome the market-based definition of Qs/Qp factors in the PEF method and develop a test-based protocol following the physical properties and future application of the recycled polymer materials.

Currently, there is a unique default value for each plastic, independent of the use that the material may have. For instance, if the LCA practitioner is performing an LCA comparing materials to produce a car bumper, then the suggested Qs/Qp ratio would be the one coming from the impact properties. If the plastic material is out of sight of the product, then it is not relevant to use optical properties. If the future application is unknown, using the overall Qs/Qp ratio is recommended; however, if it is known, then the relevant physical properties for the material performance will also be known, and a more specific ratio should be used.

Additional research for the determination of strict methods and testing conditions for the calculation of quality degradation factors for polymers is recommended to improve the accuracy of the developed LCAs of polymer products. This should be a subject for discussion within the LCA community and during the processes feeding the recommendations by the European Commission in order to determine the quality degradation factors for different kinds of polymers in the future. This research, along with other scientific efforts, can serve as a solid base for the improvement of the methods for LCA practitioners.

## Figures and Tables

**Table 1:** Calculated quality changes of the polymers based on the tensile test, maximum deformation, and Young’s modulus.

Material	Cycle	Tensile Strength (MPa)		Q Factor	Max. Deform. (%)		Q Factor	Young’s Modulus (GPa)		Q Factor
		Meas.	Calcul.		Meas.	Calcul.		Meas.	Calcul.	
HDPE	Virgin	19.7	19.528	**0.996**	10.28	10.247	**0.996**	0.967	0.949	**0.989**
	1	19.2	19.461	0.996	10.46	10.292	0.995	0.936	0.959	0.989
	2	19.39	19.394	0.993	10.49	10.336	0.991	1.024	0.969	0.979
	3	19.42	19.327	0.989	10.09	10.381	0.987	0.991	0.979	0.968
	4	19.41	19.260	0.986	10.05	10.426	0.982	1.010	0.990	0.958
	5	18.99	19.193	0.982	10.59	10.470	0.978	0.962	1.000	0.949
	6	19.1	19.126	0.979	10.57	10.515	0.974	1.060	1.010	0.939
	7	19.14	19.059	0.908	10.7	10.559	0.970	1.070	1.020	0.930
PHB	Virgin	24.7	24.482	**0.928**	7.923	6.729	**0.838**	1.780	1.894	**0.941**
	1	22.98	22.725	0.928	5.803	5.636	0.837	1.852	2.013	0.940
	2	19.07	20.967	0.856	3.096	4.542	0.675	2.330	2.132	0.888
	3	20	19.210	0.784	2.589	3.449	0.512	2.462	2.251	0.841
	4	18.26	17.452	0.712	2.714	2.355	0.350	2.407	2.370	0.799
	5	16.72	15.695	0.641	1.334	1.262	0.187	2.487	2.489	0.761
	6	12.74	13.937	0.569	0.817	0.168	0.025	2.441	2.607	0.726
PLA	Virgin	50.57	49.675	**0.648**	2.094	2.061	**0.631**	4.129	4.124	**0.999**
	1	30.41	32.200	0.648	1.237	1.301	0.631	4.111	4.120	0.998
	2	15.62	14.725	0.457	0.574	0.541	0.416	4.120	4.115	0.998
PET	Virgin	59.21	61.202	**0.945**	4.075	4.456	**0.948**	2.869	2.867	**0.997**
	1	55.17	57.837	0.945	4.041	4.225	0.948	2.852	2.875	0.997
	2	55.83	54.473	0.890	4.295	3.99	0.896	2.880	2.883	0.994
	3	54.23	51.108	0.835	4.147	3.764	0.844	2.897	2.891	0.991
	4	51.60	47.744	0.780	3.951	3.533	0.792	2.932	2.899	0.988
	5	47.17	44.379	0.725	3.334	3.302	0.741	2.938	2.907	0.986
	6	34.55	41.014	0.670	2.508	3.072	0.689	2.869	2.915	0.983

**Table 2:** Calculated quality changes of the polymers based on the Flexural Strength and Flexural modulus.

Material	Cycle	Flexural Strength (MPa)		Q Factor	Max. Deform. (%)		Q Factor	Flexural Modulus (MPa)		Q Factor
		Meas.	Calcul.		Meas.	Calcul.		Meas.	Calcul.	
HDPE	Virgin	22.960	22.913	**0.998**	10.25	10.315	**0.989**	768.6	831.682	**0.986**
	1	22.510	22.86	0.997	10.08	10.200	0.988	891.4	843.684	0.985
	2	22.870	22.814	0.995	10.06	10.085	0.977	901.8	855.686	0.971
	3	22.910	22.765	0.993	10.08	9.970	0.966	839.7	867.688	0.958
	4	23.090	22.716	0.991	10.09	9.855	0.955	898.6	879.69	0.945
	5	22.870	22.666	0.989	9.977	9.740	0.944	877.7	891.692	0.932
	6	22.030	22.617	0.987	9.257	9.625	0.933	856.2	903.694	0.920
	7	22.690	22.567	0.984	9.489	9.510	0.921	838.2	915.696	0.908
PHB	Virgin	34.290	38.783	**0.96**	10.47	10.841	**0.867**	781.1	876.76	**0.849**
	1	36.250	37.237	0.960	10.37	9.403	0.867	773.3	1033.19	0.848
	2	39.930	35.690	0.920	7.332	7.9648	0.734	1668	1189.62	0.737
	3	38.710	34.144	0.880	5.896	6.526	0.601	1543	1346.05	0.651
	4	34.610	32.598	0.840	6.163	5.088	0.469	1294	1502.48	0.583
	5	28.270	31.051	0.800	3.309	3.649	0.336	1445	1658.91	0.528
	6	26.950	29.505	0.760	2.142	2.211	0.203	1918	1815.34	0.482
PLA	Virgin	83.280	82	**0.664**	3.501	3.448	**0.65**	3660	3630.7	**0.986**
	1	51.90	54.470	0.664	2.136	2.241	0.649	3621	3679.7	0.986
	2	28.22	26.940	0.494	1.086	1.033	0.461	3758	3728.7	0.986
PET	Virgin	93.40	93.498	**0.983**	7.167	7.331	**0.971**	2474	2431.921	**0.994**
	1	89.45	91.920	0.983	7.045	7.124	0.971	2373	2444.742	0.994
	2	90.06	90.342	0.966	6.973	6.918	0.943	2436	2457.563	0.989
	3	92.23	88.764	0.949	7.053	6.711	0.915	2503	2470.384	0.984
	4	86.88	87.186	0.932	6.425	6.504	0.887	2501	2483.205	0.979
	5	89.90	85.607	0.915	6.599	6.298	0.859	2556	2496.026	0.974
	6	79.43	84.029	0.898	5.717	6.091	0.830	2450	2508.847	0.969

**Table 3:** Calculated quality changes of the polymers based on the Impact Strength.

Material	Cycle	Charpy Unnotched (kJ/m^2^)		Q Factor	Charpy Notched (kJ/m^2^)		Q Factor	Izod (J/m)		Q Factor
		Meas.	Calcul.		Meas.	Calcul.		Meas.	Calcul.	
HDPE	Virgin	no break	no break	**N/A**	14.483	15.483	**0.980**	no break	no break	**N/A**
	1	no break	no break	N/A	14.945	15.169	0.979	no break	no break	N/A
	2	no break	no break	N/A	16.056	14.855	0.959	no break	no break	N/A
	3	no break	no break	N/A	15.773	14.541	0.939	no break	no break	N/A
	4	no break	no break	N/A	13.141	14.227	0.918	no break	no break	N/A
	5	no break	no break	N/A	14.230	13.912	0.898	no break	no break	N/A
	6	no break	no break	N/A	13.623	13.598	0.878	no break	no break	N/A
	7	no break	no break	N/A	12.816	13.284	0.857	no break	no break	N/A
PHB	Virgin	no break	no break	**0.81**	13.786	14.614	**0.729**	157.869	169.386	**0.928**
	1	no break	no break		12.106	10.657	0.729	146.500	140.822	0.928
	2	42.393	38.528		6.285	6.700	0.458	132.381	112.258	0.838
	3	27.634	31.238	0.811	2.538	2.744	0.187	76.093	83.694	0.482
	4	21.358	23.949	0.622	2.538			44.804	55.130	0.283
	5	17.195	16.659	0.432	2.538			30.196	26.566	0.191
	6	11.165	9.37	0.243	2.538			30.196		
PLA	Virgin	27.508	27.508	**0.760**	1.883	1.883	**0.952**	32.376	32.366	**1**
	1	20.869	20.869	0.758	1.793	1.793	0.952	31.867	32.366	1
	2	19.922	19.922	0.954	0	0		33.053	32.366	1
PET	Virgin	no break		**0.827**	4.725	4.871	**0.860**	no break		**0.793**
	1	40.772	39.704		3.980	4.191	0.860	no break		
	2	33.388	32.820	0.826	3.544	3.512	0.720	429.357	447.696	
	3	23.379	25.937	0.653	3.469	2.832	0.581	389.883	355.122	0.793
	4	18.375	19.053	0.479	2.356	2.152	0.441	252.516	262.548	0.586
	5	13.591	12.170	0.306	0.955	1.472	0.302	159.082	169.974	0.379
	6	5.466	5.286	0.133	not possible			81.889	77.4	0.173

**Table 4:** Calculated quality changes in the polymers based on the density test.

Material	Cycle	Density (g/mL)			Q Factor
		Meas.	Dsnv.	Calcul.	
HDPE	Virgin	0.94162	0.00651	0.9467	**0.9983**
	1	0.94488	0.00933	0.9451	0.9983
	2	0.95654	0.01116	0.9435	0.9966
	3	0.93696	0.00415	0.9419	0.9949
	4	0.93619	0.00356	0.9403	0.9932
	5	0.93887	0.00534	0.9387	0.9915
	6	0.93924	0.00139	0.9371	0.9898
	7	0.93376	0.00151	0.9355	0.9881
PHB	Virgin	1.22325	0.00751	1.2175	**0.9999**
	1	1.21073	0.00315	1.2174	0.9999
	2	1.21486	0.00233	1.2173	0.9998
	3	1.22108	0.00587	1.2172	0.9997
	4	1.21309	0.00177	1.2171	0.9997
	5	1.22258	0.00403	1.2170	0.9996
	6	1.21512	0.00917	1.2169	0.9995
PLA	Virgin	1.25043	0.0039	1.2496	**0.9981**
	1	1.24594	0.0038	1.2473	0.9981
	2	1.24593	0.0084	1.2450	0.9981
PET	Virgin	1.29650	0.0061	1.3094	**0.9987**
	1	1.31411	0.0075	1.3078	0.9987
	2	1.32126	0.0074	1.3062	0.9975
	3	1.29114	0.0040	1.3046	0.9963
	4	1.30600	0.0030	1.3030	0.9951
	5	1.32489	0.0052	1.3014	0.9938
	6	1.27990	0.0024	1.2998	0.9926

**Table 5:** Calculated quality changes in the polymers based on the MFI.

Material	Cycle	MFI (g/10 min)				Q Factor
		Meas.	Dsnv.	Calcul.	Conditions	
HDPE	Virgin	5.2512	0.0447	5.219	210 °C; 2.16 kg	**0.989**
	1	5.0719	0.3115	5.160		0.988
	2	5.1742	0.1282	5.102		0.977
	3	5.0260	0.1993	5.044		0.966
	4	5.0019	0.1704	4.986		0.955
	5	4.8554	0.1088	4.928		0.944
	6	4.9997	0.1293	4.870		0.933
	7	4.7459	0.0611	4.812		0.922
PHB	Virgin	17.2097	1.7920	10.910	190 °C; 1.2 kg	**0.644–0.928**
	1	18.5470	0.5980	16.951		0.643
	2	21.7259	2.3010	22.992		0.474
	3	23.5317	6.4988	29.033		0.375
	4	31.8829	5.4546	35.074		0.311
	5	43.9876	3.1875	41.115		0.265
	6	56.6935	6.6023	47.157		0.231
PLA	Virgin	29.6922	2.6109	29.692	190 °C; 1.2 kg	**0.444**
	1	66.8335	1.4256	66.833		0.444
	2	70.1580	26.311	70.158		0.423
PET	Virgin	14.1133	0.5065	7.809	256 °C; 1.2 kg	**0.282–0.675**
	1	20.8902	1.1703	27.638		0.282
	2	45.9197	0.9473	47.467		0.164
	3	65.4140	6.4096	67.296		0.116
	4	90.9959	1.1810	87.125		0.089
	5	not possible				
	6	not possible				

**Table 6:** Calculation of the quality factor based on the maximum recycling cycles of the polymers.

Material	Max Recycling Cycles	Q Factor
HDPE	7	1 (0.875)
PHB	6	0.857
PLA	2	0.670
PET	6	0.857

**Table 7:** Calculated optical changes in the polymers based on the Cielab test.

	ΔE Measured			
	HDPE	PLA	PHB	PET
Cycle 1	1.49	1.31	1.50	3.05
Cycle 2	2.42	3.46	2.61	12.38
Cycle 3	3.15		4.81	24.01
Cycle 4	3.67		11.32	31.74
Cycle 5	4.63		14.54	37.52
Cycle 6	5.28		14.40	40.54
Cycle 7	5.56			
**Q factor**	**0.671**	**0.764**	**0.665**	**0.328**

**Table 8:** Summarized results on the quality changes of the polymers in recycling.

Properties/Material		HDPE	PHB	PLA	PET
**Mechanical**	Tensile	0.997	0.928	0.648	0.945
	Max deformation	0.996	0.838	0.631	0.948
	Young’s modulus	0.989	0.941	0.999	0.997
	Flexural Strength	0.998	0.960	0.664	0.983
	Maximum deformation	0.989	0.867	0.650	0.972
	Flexural modulus	0.986	0.849	0.987	0.995
	Charpy unnoched	N/A	0.811	0.759	0.827
	Charpy notched	0.980	0.729	0.952	0.860
	Izod	N/A	0.928	1.000	0.793
	Density	0.998	1.000	0.998	0.999
	${RQ}^{mech}$	**0.980**	**0.729**	**0.631**	**0.793**
**Processing**	MFI	0.989	0.644–0.928	0.444	0.283– 0.676
	Maximum recycling cycles	1	0.857	0.670	0.857
	${RQ}^{proc}$	**0.989**	**0.644–0.928**	**0.444**	**0.283–0.676**
**Optical**	ΔE-CieLab	0.671	0.764	0.665	0.328
	${RQ}^{opt}$	**0.671**	**0.764**	**0.665**	**0.328**
$\alpha^{rec:vir}=min\;[{RQ}^{mech},{RQ}^{opt},{RQ}^{proc}]$		**0.671**	**0.644**	**0.444**	**0.328**
**PEF default values**		**0.9**	**N/A**	**N/A**	**0.9**

## Data Availability

All testing data are publicly available in the Supplementary Materials section.

## Supplementary Materials

The following supporting information can be downloaded at: https://www.mdpi.com/article/10.3390/polym17121629/s1, Table SA1. Processing data for producing the test specimens; Figure SB1. Method for calculation of the changes of the properties of the polymers based on the trendline; Table SC1. Measured data on Tensile test and Young’s modulus; Table SD1. Measured data on the Flexural Strength test and Flexural modulus; Table SE1. Measured data on Impact Strength; Photo SG1. Problems with measuring MFI of PHB in the lab; Picture SH1. Processing problems of PHB in the 6th recycling cycle; Picture SH2. Processing problems of PET in the 6th recycling cycle; Picture SH3. Processing problems of PLA in the 1st and 2nd recycling cycle; Table SI1. Changes in the Optical properties of HDPE; Table SI2. Changes of the Optical properties of PLA; Table SI3. Changes of the Optical properties of PHB; Table SI4. Changes of the Optical properties of PET; Picture SI1. Manufactured PET bottle with disregarded optical properties, clearly stating to the customers about the compromise related to the visual quality of the bottle; Picture SI2. Difference between the optical properties of PET between the virgin and the 1st recycling cycle.

## Abbreviations

The following abbreviations are used in this manuscript:

Bio-PET	Bio-based Polyethylene Terephthalate
Bio-HDPE	Bio-based High-Density Polyethylene
EU	European Union
HDPE	High-Density Polyethylene
LCA	Life Cycle Assessment
OECD	Organization for Economic Co-operation and Development
PET	Polyethylene Terephthalate
PEF	Product Environmental Footprint
PHB	Polyhydroxybutyrate
PLA	Polylactic Acid
UNESCO	United Nations Educational, Scientific and Cultural Organization

## References

[1] OECD (2022). Global Plastics Outlook: Economic Drivers, Environmental Impacts and Policy Options.

[2] Statista Annual Production of Plastics Worldwide From 1950 to 2022. Annual Production of Plastics Worldwide from 1950 to 2022 (in Million Metric Tons). https://www.statista.com/statistics/282732/global-production-of-plastics-since-1950/#:~:text=The-worldwide-production-of-plastics,-production-has-soared-since-1950s.

[3] Geyer R., Jambeck J.R., Law K.L. (2017). Production, use, and fate of all plastics ever made. Sci. Adv..

[4] Tan J., Jia S., Ramakrishna S. (2023). Accelerating Plastic Circularity: A Critical Assessment of the Pathways and Processes to Circular Plastics. Processes.

[5] European Commission EU Calls for Agreement on Global Rules to end Plastic Pollution. https://environment.ec.europa.eu/news/eu-calls-agreement-global-rules-end-plastic-pollution-2023-05-26_en.

[6] European Commission (2020). Roadmap–New Circular Economy Action Plan.

[7] European Commission (2018). A European Strategy for Plastics in a Circular Economy.

[8] Plastic Europe Plastics—The Facts 2022. https://plasticseurope.org/knowledge-hub/plastics-the-facts-2022/.

[9] SYSTEMIQ (2022). ReShaping Plastics: Pathways to a Circular. Climate Neutral Plastics System in Europe.

[10] Sazdovski I., Bojovic D., Batlle-Bayer L., Aldaco R., Margallo M., Fullana-i-Palmer P. (2022). Circular Economy of Packaging and Relativity of Time in Packaging Life Cycle. Resour. Conserv. Recycl..

[11] Rigamonti L., Niero M., Haupt M., Grosso M., Judl J. (2018). Recycling processes and quality of secondary materials: Food for thought for waste-management-oriented life cycle assessment studies. Waste Manag..

[12] Eriksen M.K., Pivnenko K., Olsson M.E., Astrup T.F. (2018). Contamination in plastic recycling: Influence of metals on the quality of reprocessed plastic. Waste Manag..

[13] Pivnenko K., Jakobsen L.G., Eriksen M.K., Damgaard A., Astrup T.F. (2018). Challenges in Plastics Recycling.

[14] Eriksen M.K., Christiansen J.D., Daugaard A.E., Astrup T.F. (2019). Closing the loop for PET, PE and PP waste from households: Influence of material properties and product design for plastic recycling. Waste Manag..

[15] Gaduan A.N., Li J., Hill G., Wallis C., Burgstaller C., Lee K.Y. (2023). Simulating the recycling of milk bottles in the UK: Influence of blending virgin and repeatedly melt-extruded high-density polyethylene. Resour. Conserv. Recycl..

[16] Jiun Y.L., Tze C.T., Moosa U., Tawawneh M.A. (2016). Effects of recycling cycle on used thermoplastic polymer and thermoplastic elastomer polymer. Polym. Polym. Compos..

[17] MBrouwer T., Chacon F.A., van Velzen E.U.T. (2020). Effect of recycled content and rPET quality on the properties of PET bottles, part III: Modelling of repetitive recycling. Packag. Technol. Sci..

[18] Sazdovski I., Hauschild M.Z., Arfelis S., Bala A., Fullana-i-Palmer P. (2023). Short Communication: Biogenic carbon in fast-moving products: A deception or real contribution to circularity?. Environ. Adv..

[19] Abbate E., Rovelli D., Andreotti M., Brondi C., Ballarino A. (2022). Plastic packaging substitution in industry: Variability of LCA due to manufacturing countries. Procedia CIRP.

[20] de Castro B.D., de Faria P.E., Viera L.M.G., Campos Rubio C.V., Maziero R., Rodriues P.C.M., Campos Rubio J.C. (2020). Recycled Green PE Composites Reinforced with Woven and Randomly Arranged Sisal Fibres Processed by Hot Compression Moulding. Acta Technol. Agric..

[21] Delgado-Aguilar M., Julián F., Tarrés Q., Méndez J.A., Mutjé P., Espinach F.X. (2017). Bio composite from bleached pine fibers reinforced polylactic acid as a replacement of glass fiber reinforced polypropylene, macro and micro-mechanics of the Young’s modulus. Compos. Part B Eng..

[22] Delgado-Aguilar M., Puig R., Sazdovski I., Fullana-i-Palmer P. (2020). Polylactic acid/polycaprolactone blends: On the path to circular economy, substituting single-use commodity plastic products. Materials.

[23] Fazli A., Stevanovic T., Rodrigue D. (2022). Recycled HDPE/Natural Fiber Composites Modified with Waste Tire Rubber: A Comparison between Injection and Compression Molding. Polymers.

[24] Nasution H., Plaiya N.G., Haafiz M.K.M., Abdullah K., Abu Bakar S., Olaiya F.G., Mohamed A., Khalil A.H.P.S. (2021). The role of amphiphilic chitosan in hybrid nanocellulose–reinforced polylactic acid biocomposite. Polym. Adv. Technol..

[25] Saidi M.A.A., Hassan A., Wahit M.U., Choy L.J., Anuar H. (2020). Thermal, dynamic mechanical analysis and mechanical properties of polybutylene terephthalate/polyethylene terephthalate blends. J. Teknol..

[26] Shahverdi M., Seifi S., Akbari A., Mohammadi K., Shamloo A., Movahhedy M.R. (2022). Melt electrowriting of PLA, PCL, and composite PLA/PCL scaffolds for tissue engineering application. Sci. Rep..

[27] RecyClass Recycled Plastic, How to Become Certified. https://recyclass.eu/get-certified/recycled-plastic/#1.

[28] Driade Reciclabilidad de Envases y Productos. https://www.driadesm.com/.

[29] Cyclos Chira Recyclability Assessment. https://www.cyclos-htp.de/chira-1/.

[30] Gerassimidou S., Lansa P., Hahladakis J.N., Lovat E., Vanzetto S., Geueke B., Groh K.J., Muncke J., Maffini M., Martin O.V. (2022). Unpacking the complexity of the PET drink bottles value chain: A chemicals perspective. J. Hazard. Mater..

[31] Attademo M., Curi L.M., Boccooni A.P.C., Barrios C.E., Peltzer P.M., Simoniello M.F., Lajmanovich R.C., Michlig M.P., Repetti M.R., Rios J.M. (2023). Microplastics and plastic additives as contaminants of emerging concern: A multi-biomarker approach using *Rhinella arenarum* tadpoles. Environ. Adv..

[32] Braungart M., McDonough W. (2002). Cradle to Cradle: Remaking the Way We Make Things.

[33] Rigamonti L., Grosso M., Sunseri M.C. (2009). Influence of assumptions about selection and recycling efficiencies on the LCA of integrated waste management systems. Int. J. Life Cycle Assess..

[34] Gala A.B., Raugei M., Fullana-i-Palmer P. (2015). Introducing a new method for calculating the environmental credits of end-of-life material recovery in attributional LCA. Int. J. Life Cycle Assess..

[35] Niero M., Negrelli A.J., Hoffmeyer S.B., Olsen S.I., Birkved M. (2016). Closing the loop for aluminum cans: Life Cycle Assessment of progression in Cradle-to-Cradle certification levels. J. Clean. Prod..

[36] (2012). Environmental Management—Life Cycle Assessment—Illustrative Examples on How to Apply ISO 14044 to Goal and Scope Definition and Inventory Analysis.

[37] European Commission Environmental Footprint Methods. https://green-business.ec.europa.eu/environmental-footprint-methods_en.

[38] (2006). Environmental Management—Life Cycle Assessment—Requirements and Guidelines.

[39] European Commission (2021). Commission Recommendation of 16.12.2021 on the use of the Environmental Footprint methods to measure and communicate the life cycle environmental performance of products and organisations. Off. J. Eur. Union C.

[40] European Commission Environmental Footprint Reference Packages, Annex C. https://eplca.jrc.ec.europa.eu/LCDN/developerEF.html.

[41] Rigamonti L., Taelman S.E., Huysveld S., Sfez S., Ragaert K., Dewulf J. (2020). A step forward in quantifying the substitutability of secondary materials in waste management life cycle assessment studies. Waste Manag..

[42] Garcia E.S., Huysveld S., Nhu T.T., De Meester S., Dewulf J. (2023). Technical substitutability of recycled materials in life cycle Assessment: A comprehensive review and framework for quantification. Waste Manag..

[43] Tonini D., Albizzati P.F., Caro D., De Meester S., Garbarino E., Blengini G.A. (2022). Quality of recycling: Urgent and undefined. Waste Manag..

[44] Steinmann Z.J.N., Huijbregts M.A.J., Reijnders L. (2018). How to define the quality of materials in a circular economy?. Resour. Conserv. Recycl..

[45] Barbato P.M., Olsson E., Rigamonti L. (2023). Quality degradation in glass recycling: Substitutability model proposal. Waste Manag..

[46] Huysveld S., Ragaert K., Demets R., Nhu T.T., Civancik-Uslu D., Kusenberg M., van Geem K.M., de Meester S., Dewulf J. (2022). Technical and market substitutability of recycled materials: Calculating the environmental benefits of mechanical and chemical recycling of plastic packaging waste. Waste Manag..

[47] Roosen M., Tonini D., Albizzati P.F., Caro D., Cristobal J., Lase I.S., Ragaert K., Dumoulin A., de Meester S. (2023). Operational Framework to Quantify ‘Quality of Recycling’ across Different Material Types. Environ. Sci. Technol..

[48] Vadenbo C., Hellweg S., Astrup T.F. (2017). Let’s Be Clear(er) about Substitution: A Reporting Framework to Account for Product Displacement in Life Cycle Assessment. J. Ind. Ecol..

[49] Demets R., Van Kets K., Huysveld S., Dewulf J., De Meester S., Ragaert K. (2021). Addressing the complex challenge of understanding and quantifying substitutability for recycled plastics. Resour. Conserv. Recycl..

[50] Schulte A., Velarde P.Á.S., Marbach L., Mörbitz P. (2023). Measuring the circularity potential of recycled LDPE based on quantity and quality conservation–A functional requirement matrix approach. Resour. Conserv. Recycl. Adv..

[51] Rosenboom J.G., Langer R., Traverso G. (2022). Bioplastics for a circular economy. Nat. Rev. Mater..

[52] (2013). Standard Test Method for Melt Flow Rates of Thermoplastics by Extrusion Plastometer.

[53] (2019). Plastics—Methods for Determining the Density of Non-Cellular Plastics—Part 1: Immersion Method, Liquid Pyknometer Method and Titration Method.

[54] (2023). Plastics—Determination of Charpy Impact Properties.

[55] (2025). Standard Test Methods for Determining the Izod Pendulum Impact Resistance of Plastics.

[56] (2019). Plastics—Determination of Tensile Properties.

[57] (2019). Plastics—Determination of Flexural Properties.

[58] (2022). Standard Practice for Calculation of Color Tolerances and Color Differences from Instrumentally Measured Color Coordinates.

[59] Pinheiro L.A., Chinelatto M.A., Canevarolo S.V. (2004). The role of chain scission and chain branching in high density polyethylene during thermo-mechanical degradation. Polym. Degrad. Stab..

[60] Haudin J.-M., Boyer S.A.E. (2017). Crystallization of Polymers in Processing Conditions: An Overview. Int. Polym. Process..

[61] Camacho W., Karlsson S. (2002). Assessment of thermal and thermo-oxidative stability of multi-extruded recycled PP, HDPE and a blend thereof. Polym. Degrad. Stab..

[62] Johnson A.M., Johnson J.A. (2023). Thermally Robust yet Deconstructable and Chemically Recyclable High-Density Polyethylene (HDPE)-Like Materials Based on Si−O Bonds. Angew. Chem. Int. Ed..

[63] Fiorillo C., Trossaert L., Bezeraj E., Debrie S., Ohnmacht H., Van Steenberge P.H.M., D′hooge D.R., Edeleva M. (2024). Molecular and material property variations during the ideal degradation and mechanical recycling of PET. RSC Sustain..

[64] Del Mar Castro López M., Pernas A.I.A., López M.J.A., Latorre A.L., Vilariño J.M.L., Rodríguez M.V.G. (2014). Assessing changes on poly(ethylene terephthalate) properties after recycling: Mechanical recycling in laboratory versus postconsumer recycled material. Mater. Chem. Phys..

[65] Muringayil J.T., Azat S., Ahmadi Z., Jazani O.M., Esmaeili A., Kianfar E., Haponiuk J., Thomas S. (2024). Polyethylene terephthalate (PET) recycling: A review. Case Stud. Chem. Environ. Eng..

[66] Beltrán F.R., Lorenzo V., de la Orden M.U., Martínez-Urreaga J. (2016). Effect of different mechanical recycling processes on the hydrolytic degradation of poly(L-lactic acid). Polym. Degrad. Stab..

[67] De Silva C.P., Menezes L.R., da Silva P.S.R.C., Tavares M.I.B. (2021). Evaluation of thermal properties of zirconium–PHB composites. J. Therm. Anal. Calorim..

[68] Olejnik O., Masek A., Zawadziłło J. (2021). Processability and mechanical properties of thermoplastic polylactide/polyhydroxybutyrate (PLA/PHB) bioblends. Materials.

[69] Farias N.C., Major I., Devine D., Fournet M.B., Pezzoli R., Taghinezhad S.F., Hesabi M. (2022). Multiple recycling of a PLA/PHB biopolymer blend for sustainable packaging applications: Rheology-morphology, thermal, and mechanical performance analysis. Polym. Eng. Sci..

[70] Rivas L.F., Casarin S.A., Nepomuceno N.C., Alencar M.I., Agnelli J.A. (2017). Reprocessability of PHB in extrusion: ATR-FTIR, tensile tests and thermal studies. Polimeros.

[71] ASM International (2004). Tensile Testing.

[72] Barnetson A. (1996). Plastic Materials for Packaging.

[73] Brewis D.M., Briggs D. (2013). Mechanical Properties and Testing of Polymers, An A–Z Reference.

[74] Perkins W.G. (1999). Polymer toughness and impact resistance. Polym. Eng. Sci..

[75] Kholodovych V., Welsh W.J. (2007). Densities of Amorphous and Crystalline Polymers. Physical Properties of Polymers Handbook.

[76] Hossain S. (2019). Optical Properties of Polymers and Their Applications.

[77] Melgosa M. (1999). Testing CIELAB-based color-difference formulas. Color Res. Appl..

