# Supplementary material for: Circular Quality of Polymers: Test-Based Evidence for Comparison of Bio-Based and Fossil-Based Polymers
Source: Polymers (Basel). 2025 Jun 12;17(12):1629. doi: 10.3390/polym17121629 (PMC12196982; doi:10.3390/polym17121629)
Supplement: Supplementary file 1 [file polymers-17-01629-s001.zip › polymers-3674475-supplementary.pdf]

Supplementary Materials

Section SA. Details related to the testing and processing equipment and processing parameters

Sample Size:

Test specimens were obtained by injection molding according to ASTM D638 Type I (dumbbell).

i) Specimens for Tensile tests and Young´s Modulus:

Length: 165 mm, Thickness: 3.2 mm, Width: up to 14 mm;

ii) Specimens for Flexural and Impact tests:

Length: 130 mm, Thickness: 3.2 mm, Width: 13 mm;

iii) Specimens for Optical tests:

Disc with a diameter of 50 mm, Thickness: 3.2 mm.

Equipment used:

i) Flexural and tensile properties testing equipment: Instron TM 1122. Load cell: 5 kN. 3 bendpoint test, 2mm per minute;

ii) Young modulus testing equipment: we used an extensometer and tests were performed until 0.25% strain on the stress-strain plot. Extensometer reference: Walter+bai AG MFA2;

iii) Charpy and IZOD testing equipment: ISO 179, CEAST RESIL 5.5;

iv) MFI testing equipment: CEAST testing equipment (7082,000, series 17959, Pianezza TO, Italy);

v) CIELAB Optical testing equipment: Technibrite Eric 950;

vi) Moulding equipment: Arburg Allrouder 220M/350-90;

vii) Shreder: SM 100 model, Retsch GmbH;

Processing parameters:

The starting processing parameters were set based on the recommendations of the polymer manufacturer and the data stated in the technical sheets, especially the melting temperature, typical moulding temperature, and drying conditions (time and temperature). Due to deterioration of the processing quality of the polymer material, after each cycle, the processing parameters were slightly adjusted in order to provide satisfactory quality of the specimens.

Table SA1. Processing data for producing the test specimens

**Material**	**Cycle**	**Moulding Temperature (°C)**						**Moulding / cooling time (s)**
		**T_1**	**T_2**	**T_3**	**T_4**	**T_5**	**T_{mold}**	
**HDPE**	Virgin	175	180	185	190	190	ambiental	40
	1							
	2							
	3							
	4							
	5							
	6							
	7							
**PHB**	Virgin	170	170	175	180	185	ambiental	
	1							
	2	165	165	170	175	180		

	3							40
	4							
	5							
	6							
PLA	Virgin	175	180	185	190	190	ambiental	50
	1				185	180		
	2			180	180	175		
PET	Virgin	260	270	280	283	293	65	60
	1							
	2							
	3							
	4							
	5							
	6							

Section SB. Method for calculation of trendline data

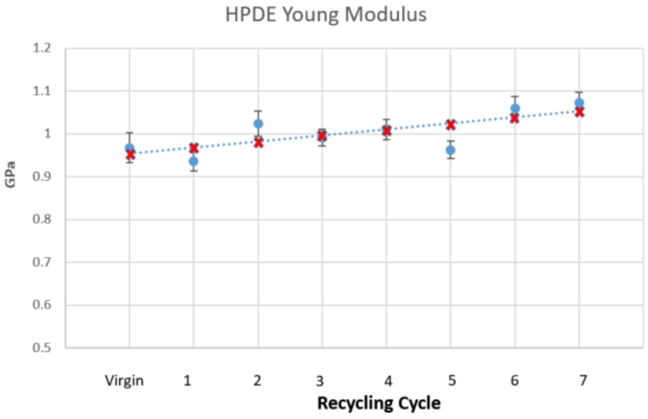

Material	Cycle	Young's modulus (GPa)	Desv.
HDPE	Virgin	0.9674	0.03444
	1	0.9363	0.02359
	2	1.024	0.02948
	3	0.9911	0.01957
	4	1.01	0.02311
	5	0.9625	0.02052
	6	1.06	0.0272
	7	1.072	0.0254

Figure SB1. Method for calculation of the changes of the properties of the polymers based on the trendline. Blue dots represent measured values based on the measuring standards and red "x"s present the calculated values

Section SC. Tensile test and Young's modulus

Table SC1. Measured data on Tensile test and Young's modulus

Material	Cycle	Tensile test						Young's modulus (GPa)	Desv.
		Tensile strength (MPa)	Desv.	Maximum deformation (%)	Desv.	Deformation at break (%)	Desv.		
HDPE	Virgin	19.7	0.314	10.28	0.377	no break (100mm elongation)	N/A	0.9674	0.03444
	1	19.2	0.229	10.46	0.174		N/A	0.9363	0.02359
	2	19.39	0.376	10.49	0.295		N/A	1.024	0.02948
	3	19.42	0.288	10.09	0.292		N/A	0.9911	0.01957
	4	19.41	0.221	10.05	0.18		N/A	1.01	0.02311
	5	18.99	0.17	10.59	0.318		N/A	0.9625	0.02052
	6	19.1	0.262	10.57	0.244		N/A	1.06	0.0272
	7	19.14	0.223	10.7	0.282		N/A	1.072	0.0254

**PHB**	Virgin	24.7	0.609	7.947	0.458	7.923	0.444	1.78	0.05102
	1	22.98	0.884	5.793	0.441	5.803	0.449	1.852	0.1072
	2	19.07	1.578	3	0.299	3.096	0.325	2.33	0.08319
	3	20	0.38	2.558	0.145	2.589	0.163	2.462	0.0331
	4	18.26	0.47	2.697	0.144	2.714	0.147	2.407	0.06909
	5	16.72	1.441	1.334	0.221	1.334	0.221	2.487	0.05962
	6	12.74	2.341	0.815	0.246	0.817	0.249	2.441	0.06054
**PLA**	Virgin	50.57	0.679	2.094	0.7	2.095	0.069	4.129	0.05781
	1	30.41	8.41	1.237	0.077	1.238	0.077	4.111	0.05452
	2	15.62	7.787	0.574	0.308	0.579	0.312	4.12	0.06125
**PET**	Virgin	59.21	0.368	4.075	0.071	no break (100mm)	N/A	2.869	0.07387
	1	55.17	1.073	4.041	0.138	22.38	23.09	2.852	0.04359
	2	55.83	2.423	4.295	0.313	23.52	24.14	2.88	0.05821
	3	54.23	2.44	4.147	0.359	8.165	10	2.897	0.03769
	4	51.6	2.391	3.951	0.238	3.595	0.235	2.932	0.02227
	5	47.17	6.864	3.334	0.489	3.338	0.489	2.938	0.04562
	6	34.55	4.072	2.508	0.29	2.508	0.29	2.869	0.02132

Section SD. Flexural test

Table SD1. Measured data on the Flexural Strength test and Flexural modulus

**Material**	**Cycle**	**Flexural test**					
		**Flexural strength (MPa)**	**Desv.**	**Maximum deformation (mm)**	**Desv.**	**Flexural modulus (MPa)**	**Desv.**
**HDPE**	Virgin	22.96	0.769	10.25	0.219	768.6	117.6
	1	22.51	0.374	10.08	0.227	891.4	81.05
	2	22.87	0.297	10.06	0.184	901.8	66.45
	3	22.91	0.79	10.08	0.189	839.7	118.3
	4	23.09	0.485	10.09	0.145	898.6	82.86
	5	22.87	0.507	9.977	0.2	877.7	69.07
	6	22.03	0.679	9.257	0.193	856.2	81.24
	7	22.69	0.83	9.489	0.287	838.2	94.25
**PHB**	Virgin	34.29	0.939	10.47	0.51	781.1	144
	1	36.25	0.632	10.37	0.342	773.3	285.6
	2	39.93	0.973	7.332	0.52	1668	157.3
	3	38.71	0.666	5.896	0.313	1543	204.9
	4	34.61	0.68	6.163	0.312	1294	259.4
	5	28.27	2.306	3.309	0.547	1445	227.1
	6	26.95	3.434	2.142	0.54	1918	414.9
**PLA**	Virgin	83.28	4.506	3.501	0.244	3660	190.2
	1	51.9	6.061	2.136	0.213	3621	140.1
	2	28.22	6.398	1.086	0.241	3758	149.6
**PET**	Virgin	93.4	1.42	7.167	0.139	2474	68.82

	1	89.45	1.77	7.045	0.233	2373	145.1
	2	90.06	1.434	6.973	0.099	2436	121.2
	3	92.23	1.398	7.053	0.162	2503	80.5
	4	86.88	8.131	6.425	1.079	2501	79.61
	5	89.9	5.184	6.5996	0.865	2556	52.56
	6	79.43	8.225	5.717	1.163	2450	119.7

Section SE: Impact Strength

Table SE1. Measured data on Impact Strength

Material	Cycle	Impact test					
		**Charpy unnotched (kJ/m2)**	**Desv.**	**Charpy notched (kJ/m2)**	**Desv.**	**Izod (J/m)**	**Desv.**
HDPE	Virgin	no break	N/A	14.48366667	2.15879	no break	N/A
	1	no break	N/A	14.9458	1.3794	no break	N/A
	2	no break	N/A	16.056	0.17494	no break	N/A
	3	no break	N/A	15.773	0.522	no break	N/A
	4	no break	N/A	13.1413	2.063	no break	N/A
	5	no break	N/A	14.2308	0.9717	no break	N/A
	6	no break	N/A	13.6239	0.6352	no break	N/A
	7	no break	N/A	12.816	1.7749	no break	N/A
PHB	Virgin	no break	N/A	13.7865	1.1753	157.86925	28.2345
	1	no break	N/A	12.1063	0.8155	146.500444	17.9047
	2	42.3935	6.06862	6.28525	0.68525	132.381375	10.3448
	3	27.634	2.85005	2.301714286	1.14468	76.0936	10.0566
	4	21.35842857	1.83223	3.413666667	0.9294	44.80433333	9.59437
	5	17.19514286	1.0625	2.1655	0.6699	30.0682	3.74813
	6	11.165	1.96414	2.184833333	0.7258	30.30416667	1.22888
PLA	Virgin	27.508	0.384	1.88322222	0.43484	32.37628571	3.45497
	1	20.86966	2.6211	1.7933	0.4989	31.86714286	3.25403
	2	19.922	4.14596	0	0	33.0532	1.21301
PET	Virgin	no break	N/A	4.725555556	2.30485	no break	N/A
	1	40.77285714	13.0802	3.980571429	0.96125	no break	N/A
	2	33.388375	10.9815	3.544375	0.64029	429.357	161.426
	3	23.3797	8.55383	3.469888889	1.09217	389.88325	71.6493
	4	18.3759	10.9334	2.35625	0.52501	252.516	41.6227
	5	13.5915	5.641	0.955777778	0.51182	159.082	24.6026
	6	5.466777778	1.67134	Not possible		81.88925	11.8366

Section SF. Statistical analysis of the measured data

1. **Anova: Single Factor analysis PHB IZOD test**

SUMMARY

Groups	Count	Sum	Average	Variance
Cycle No5	6	150.341	30.0682	14.0484907
Cycle No6	7	181.825	30.30416667	1.510148167

ANOVA

Source of Variation	SS	df	MS	F	P-value	F crit
Between Groups	0.151855276	1	0.151855276	0.021440173	0.886813514	5.117355029
Within Groups	63.74470363	9	7.082744848			
Total	63.89655891	10				

2. **Anova: Single Factor analysis PHB Charpy Notched test**

SUMMARY

Groups	Count	Sum	Average	Variance
Cycle 3	7	16.112	2.301714286	1.310290238
Cycle 4	6	20.482	3.413666667	0.863793067
Cycle 5	4	8.662	2.1655	0.448764333
Cycle 6	6	13.109	2.184833333	0.526791767

ANOVA

Source of Variation	SS	df	MS	F	P-value	F crit
Between Groups	6.294950883	3	2.098316961	2.466934249	0.09341872	3.127350005
Within Groups	16.1609586	19	0.850576768			
Total	22.45590948	22				

3. **Anova: Single Factor analysis PLA IZOD test**

SUMMARY

Groups	Count	Sum	Average	Variance
Cycle 0	7	226.634	32.37628571	11.9368479
Cycle 1	7	223.07	31.86714286	10.58871614
Cycle 2	5	165.266	33.0532	1.4713827

ANOVA

Source of Variation	SS	df	MS	F	P-value	F crit
Between Groups	4.103955441	2	2.05197772	0.232784289	0.794961333	3.633723468
Within Groups	141.0389151	16	8.814932193			
Total	145.1428705	18				

Section SG. Problems with testing of MFI

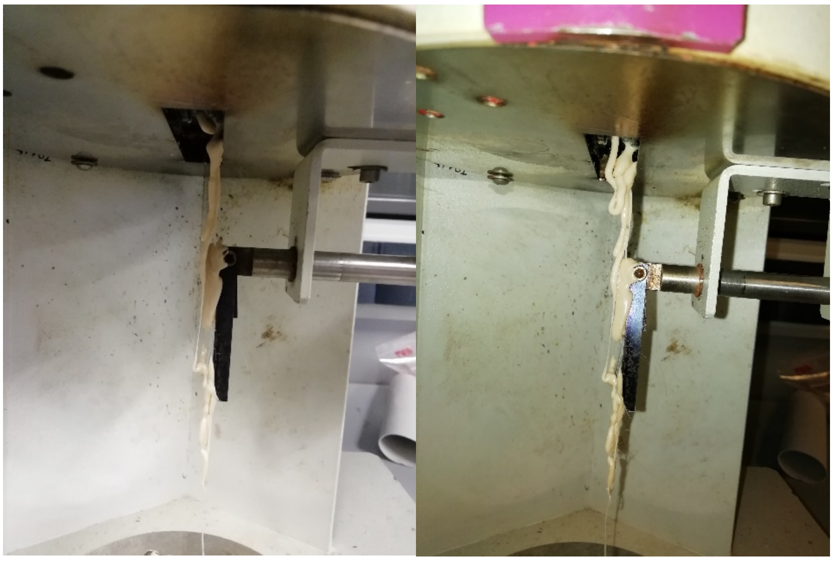

Photo SG1. Problems with measuring MFI of PHB in the lab

Section SH. Processing problems of tested polymers

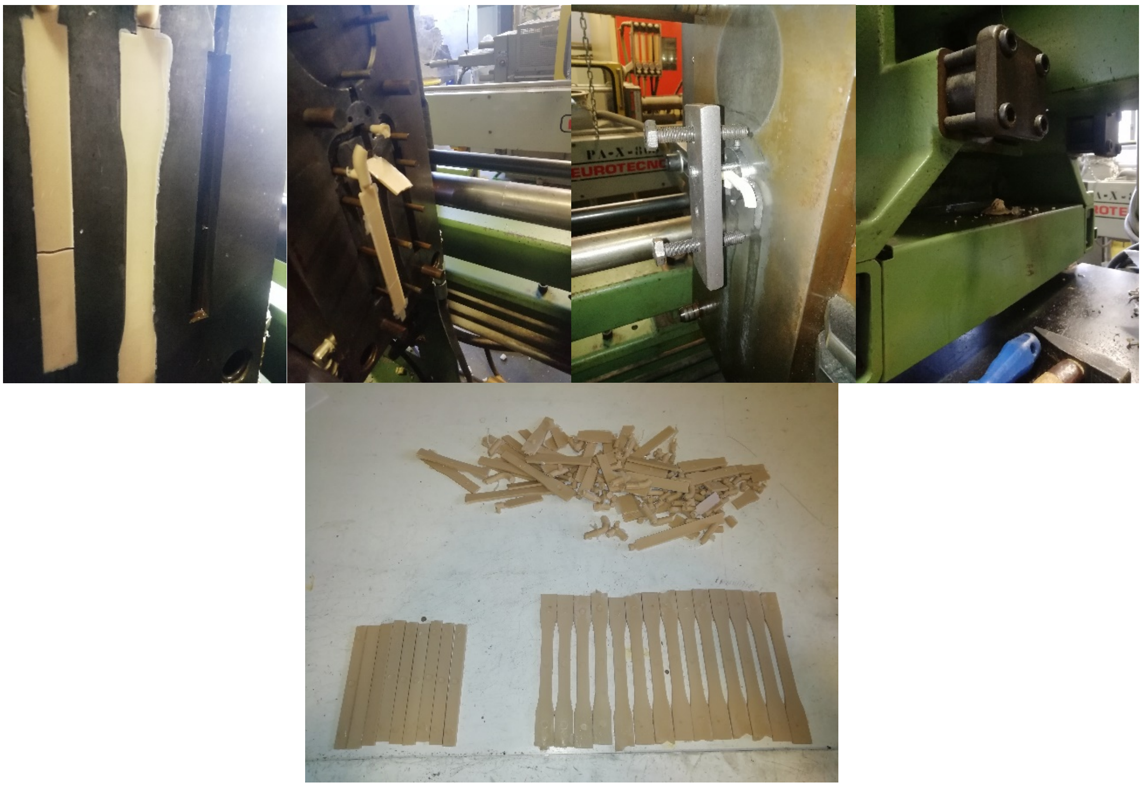

Picture SH1. Processing problems of PHB in the 6th recycling cycle

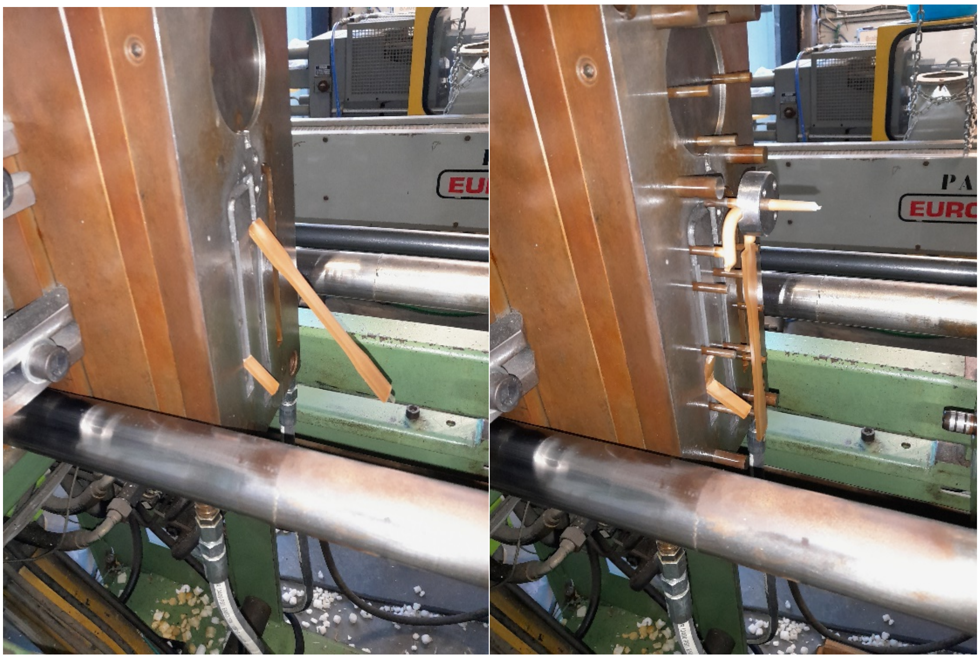

Picture SH2. Processing problems of PET in the 6th recycling cycle

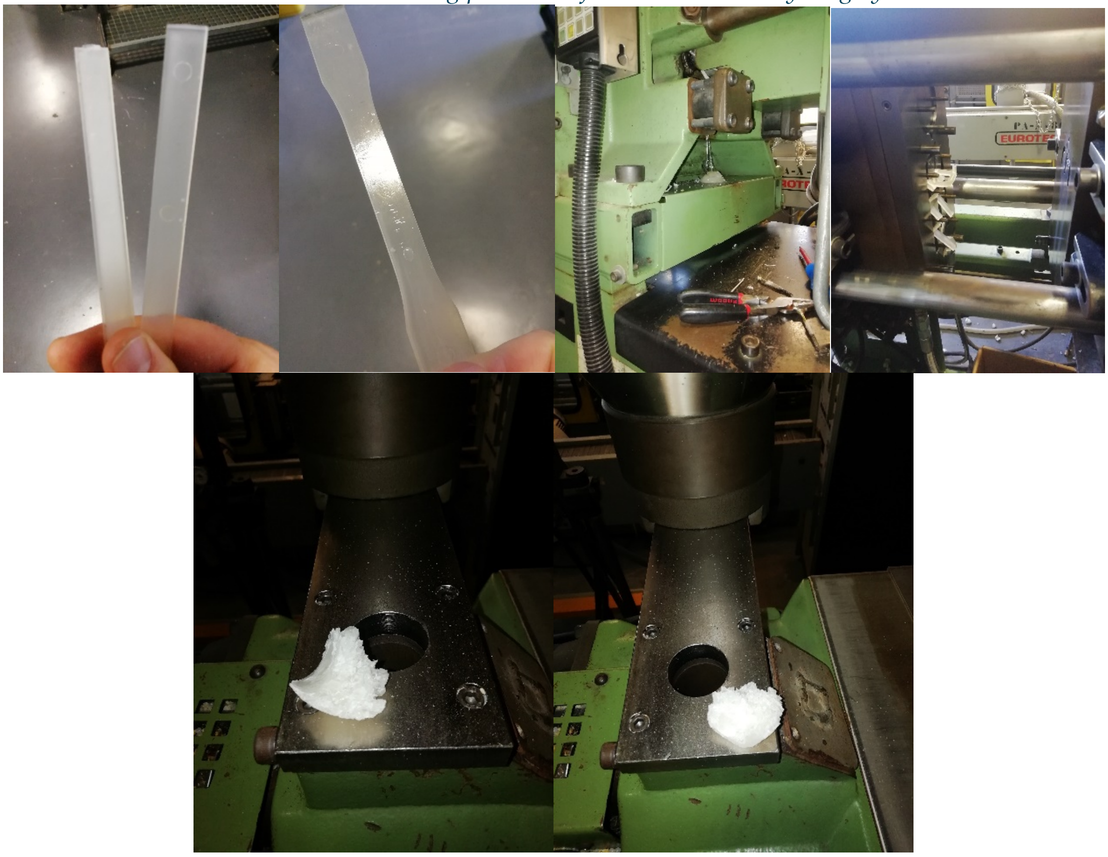

Picture SH3. Processing problems of PLA in the 1st and 2nd recycling cycle

Section SI. Optical properties

Table SI1. Changes in the Optical properties of HDPE

	HDPE Virgin								
	**R (x)**	**R (y)**	**R (z)**	**L**	**a**	**b**	**L***	**a***	**b***
**M 1**	41.26	42.57	42.86	65.24	-2.64	-0.31	71.26	-2.91	-0.35
**M 2**	41.49	42.79	43.05	65.41	-2.64	-0.28	71.41	-2.91	-0.31

**M 3**	41.74	43.03	43.41	65.60	-2.53	-0.41	71.57	-2.79	-0.44
**M 4**	41.39	42.72	43.12	65.36	-2.66	-0.42	71.37	-2.93	-0.47
**M 5**	41.89	43.18	43.8	65.72	-2.44	-0.65	71.68	-2.69	-0.71
**Mitjana**	41.55	42.86	43.25	65.47	-2.58	-0.41	71.46	-2.85	-0.46
**Desv.**	0.2574	0.2449	0.3664	0.1923	0.0944	0.1454	0.1666	0.1033	0.1561

	**HDPE cycle 1**								
	**R (x)**	**R (y)**	**R (z)**	**L**	**a**	**b**	**L***	**a***	**b***
**M 1**	42.54	43.57	43.04	66.01	-2.47	0.57	71.94	-2.72	0.63
**M 2**	41.52	42.60	41.96	65.27	-2.66	0.69	71.28	-2.94	0.76
**M 3**	42.01	43.03	41.99	65.6	-2.75	1.11	71.58	-3.03	1.22
**M 4**	41.82	42.8	41.71	65.42	-2.70	1.17	71.42	-2.98	1.30
**M 5**	41.99	42.98	41.93	65.56	-2.69	1.12	71.54	-2.96	1.24
**Mitjana**	41.98	43.00	42.13	65.57	-2.65	0.93	71.55	-2.93	1.03
**Desv.**	0.3714	0.3627	0.5227	0.2771	0.1078	0.2799	0.2464	0.1199	0.3106

	**HDPE cycle 2**								
	**R (x)**	**R (y)**	**R (z)**	**L**	**a**	**b**	**L***	**a***	**b***
**M 1**	41.02	41.88	40.45	64.71	-2.64	1.54	70.79	-2.92	1.72
**M 2**	41.71	42.54	40.91	65.22	-2.68	1.76	71.24	-2.96	1.95
**M 3**	40.89	41.72	39.81	64.59	-2.86	2.07	70.68	-3.17	2.32
**M 4**	41.30	42.18	40.41	64.94	-2.84	1.90	70.99	-3.15	2.12
**M 5**	41.4	42.27	41.07	65.01	-2.51	1.29	71.05	-2.77	1.43
**Mitjana**	41.26	42.12	40.53	64.89	-2.71	1.71	70.95	-2.99	1.91
**Desv.**	0.3233	0.3241	0.4938	0.2491	0.1459	0.3056	0.2203	0.1674	0.3465

	**HDPE cycle 3**								
	**R (x)**	**R (y)**	**R (z)**	**L**	**a**	**b**	**L***	**a***	**b***
**M 1**	39.34	40.16	39.03	63.37	-2.44	1.25	69.58	-2.72	1.40
**M 2**	38.86	39.63	38.01	62.95	-2.63	1.80	69.21	-2.94	2.03
**M 3**	38.84	39.64	37.89	62.96	-2.78	1.95	69.22	-3.11	2.20
**M 4**	38.62	39.42	38.00	62.79	-2.59	1.59	69.06	-2.90	1.79
**M 5**	38.63	39.46	38.29	62.82	-2.51	1.30	69.08	-2.81	1.46
**Mitjana**	38.86	39.66	38.24	62.98	-2.59	1.58	69.23	-2.90	1.78
**Desv.**	0.2921	0.2953	0.4636	0.2319	0.1290	0.3052	0.2088	0.1467	0.3485

	**HDPE cycle 4**								
	**R (x)**	**R (y)**	**R (z)**	**L**	**a**	**b**	**L***	**a***	**b***
**M 1**	39.32	40.00	37.94	63.25	-2.66	2.28	69.47	-2.97	2.57
**M 2**	39.42	40.10	37.73	63.33	-2.82	2.62	69.54	-3.15	2.96
**M 3**	38.85	39.51	37.34	62.85	-2.67	2.41	69.12	-2.99	2.73
**M 4**	39.25	39.91	37.86	63.17	-2.61	2.27	69.40	-2.91	2.57

**M 5**	39.11	39.85	38.27	63.13	-2.53	1.75	69.36	-2.83	1.97
**Mitjana**	39.19	39.87	37.83	63.15	-2.66	2.27	69.38	-2.97	2.56
**Desv.**	0.2210	0.2243	0.3379	0.1824	0.1062	0.3211	0.1597	0.1183	0.3665

	**HDPE cycle 5**								
	**R (x)**	**R (y)**	**R (z)**	**L**	**a**	**b**	**L***	**a***	**b***
**M 1**	38.05	38.63	36.45	62.16	-2.55	2.46	68.49	-2.86	2.80
**M 2**	38.06	38.64	36.21	62.16	-2.69	2.73	68.49	-3.02	3.12
**M 3**	38.18	38.77	36.24	62.26	-2.76	2.84	68.58	-3.10	3.24
**M 4**	38.22	38.75	36.20	62.25	-2.63	2.87	68.57	-2.96	3.27
**M 5**	37.79	38.35	36.03	61.92	-2.57	2.62	68.28	-2.89	2.99
**Mitjana**	38.06	38.628	36.226	62.15	-2.64	2.70	68.48	-2.97	3.08
**Desv.**	0.1681	0.1677	0.1498	0.1371	0.0866	0.1683	0.1207	0.0974	0.1935

	**HDPE cycle 6**								
	**R (x)**	**R (y)**	**R (z)**	**L**	**a**	**b**	**L***	**a***	**b***
**M 1**	38.31	38.72	36.05	62.23	-2.44	3.00	68.55	-2.74	3.43
**M 2**	38.03	38.48	35.62	62.03	-2.63	3.22	68.37	-2.96	3.69
**M 3**	38.66	39.04	35.80	62.48	-2.70	3.63	68.78	-3.02	4.16
**M 4**	37.66	38.11	35.00	61.73	-2.79	3.53	68.10	-3.14	4.06
**M 5**	37.71	38.17	35.20	61.78	-2.73	3.36	68.14	-3.08	3.86
**Mitjana**	38.07	38.50	35.53	62.05	-2.66	3.35	68.39	-2.99	3.84
**Desv.**	0.4198	0.3877	0.4304	0.3134	0.1348	0.2501	0.2849	0.1540	0.2923

	**HDPE cycle 7**								
	**R (x)**	**R (y)**	**R (z)**	**L**	**a**	**b**	**L***	**a***	**b***
**M 1**	38.38	38.57	35.65	62.11	-2.09	3.29	68.44	-2.34	3.77
**M 2**	38.31	38.51	35.28	62.05	-2.28	3.64	68.39	-2.56	4.18
**M 3**	37.61	37.85	34.57	61.52	-2.43	3.73	67.91	-2.73	4.3
**M 4**	38.23	38.48	35.19	62.03	-2.42	3.71	68.37	-2.72	4.26
**M 5**	38.81	39	35.67	62.45	-2.3	3.73	68.75	-2.58	4.28
**Mitjana**	38.27	38.48	35.27	62.03	-2.30	3.62	68.37	-2.59	4.16
**Desv.**	0.4308	0.4112	0.4475	0.3329	0.1376	0.1881	0.3005	0.1581	0.2216

Table SI2. Changes of the Optical properties of PLA

	**PLA Virgin**								
	**R (x)**	**R (y)**	**R (z)**	**L**	**a**	**b**	**L***	**a***	**b***
**M 1**	6.97	7.36	8.75	27.13	-0.20	-3.59	32.61	-0.29	-4.98
**M 2**	6.94	7.32	8.68	27.05	-0.17	-3.53	32.51	-0.25	-4.91
**M 3**	6.92	7.28	8.66	26.98	-0.08	-3.58	32.44	-0.12	-4.98
**M 4**	7.07	7.42	8.77	27.23	-0.02	-3.49	32.73	-0.02	-4.84
**M 5**	7.11	7.47	8.96	27.34	0.03	-3.80	32.86	0.05	-5.25

**Mitjana**	7.00	7.37	8.76	27.15	-0.09	-3.60	32.63	-0.13	-4.99
**Desv.**	0.0835	0.0762	0.1189	0.1429	0.0973	0.1199	0.1687	0.1454	0.1555

	**PLA cycle 1**								
	**R (x)**	**R (y)**	**R (z)**	**L**	**a**	**b**	**L***	**a***	**b***
**M 1**	6.89	7.2	8.21	26.83	-0.28	-2.64	32.25	-0.41	-3.73
**M 2**	6.99	7.27	8.27	26.97	-0.19	-2.58	32.42	-0.28	-3.65
**M 3**	6.91	7.22	8.30	26.88	-0.24	-2.81	32.31	-0.35	-3.96
**M 4**	7.01	7.3	8.28	27.01	-0.20	-2.54	32.47	-0.29	-3.59
**M 5**	6.95	7.26	8.27	26.94	-0.28	-2.64	32.38	-0.41	-3.72
**Mitjana**	6.95	7.25	8.27	26.93	-0.24	-2.64	32.37	-0.35	-3.73
**Desv.**	0.0510	0.0400	0.0336	0.0716	0.0427	0.1031	0.0873	0.0626	0.1405

	**PLA cycle 2**								
	**R (x)**	**R (y)**	**R (z)**	**L**	**a**	**b**	**L***	**a***	**b***
**M 1**	7.79	7.92	8.24	28.15	-0.28	-0.78	33.82	-0.40	-1.12
**M 2**	7.57	7.81	8.39	27.94	-0.47	-1.46	33.58	-0.69	-2.08
**M 3**	7.63	7.87	8.50	28.05	-0.42	-1.56	33.71	-0.61	-2.21
**M 4**	7.83	8.01	8.54	28.31	-0.25	-1.31	34.01	-0.36	-1.86
**M 5**	7.61	7.81	8.33	27.95	-0.35	-1.31	33.59	-0.50	-1.86
**M 6**	7.58	7.78	8.13	27.89	-0.54	-0.88	33.52	-0.78	-1.27
**Mitjana**	7.67	7.87	8.36	28.05	-0.39	-1.22	33.71	-0.56	-1.73
**Desv.**	0.1125	0.0864	0.1555	0.1583	0.1122	0.3158	0.1840	0.1655	0.4405

Table SI3. Changes of the Optical properties of PHB

	**PHB cycle 0**								
	**R (x)**	**R (y)**	**R (z)**	**L**	**a**	**b**	**L***	**a***	**b***
**M 1**	71.46	67.13	50.46	81.93	0.21	14.24	85.57	0.23	15.89
**M 2**	71.69	67.41	50.87	82.10	0.18	14.1	85.71	0.19	15.71
**M 3**	71.38	66.98	50.3	81.84	0.33	14.27	85.49	0.35	15.93
**M 4**	71.78	67.49	51.11	82.15	0.26	13.96	85.75	0.28	15.53
**M 5**	71.71	67.45	51.2	82.13	0.28	13.85	85.73	0.30	15.40
**Mitjana**	71.60	67.29	50.79	82.03	0.25	14.08	85.65	0.27	15.69
**Desv.**	0.1736	0.2245	0.3956	0.1373	0.0589	0.1798	0.1140	0.0620	0.2279

	**PHB cycle 1**								
	**R (x)**	**R (y)**	**R (z)**	**L**	**a**	**b**	**L***	**a***	**b***
**M 1**	68.95	64.63	48.59	80.40	0.48	13.97	84.29	0.50	15.68
**M 2**	68.50	64.10	47.94	80.06	0.57	14.13	84.02	0.59	15.92
**M 3**	68.68	64.23	48.22	80.14	0.72	13.98	84.09	0.75	15.72
**M 4**	68.57	64.2	48.51	80.12	0.73	13.70	84.07	0.75	15.38
**M 5**	69.27	65.03	49.41	80.64	0.52	13.56	84.5	0.54	15.16

**Mitjana**	68.79	64.44	48.53	80.27	0.60	13.87	84.19	0.63	15.57
**Desv.**	0.3164	0.3878	0.5528	0.2436	0.1150	0.2317	0.1996	0.1176	0.3005

	**PHB cycle 2**								
	**R (x)**	**R (y)**	**R (z)**	**L**	**a**	**b**	**L***	**a***	**b***
**M 1**	67.58	63.60	49.27	79.75	0.65	12.58	83.76	0.67	14.03
**M 2**	67.11	63.04	48.51	79.39	0.73	12.80	83.46	0.76	14.33
**M 3**	66.67	62.50	47.69	79.06	0.78	13.11	83.18	0.81	14.74
**M 4**	67.19	63.11	48.39	79.44	0.65	12.97	83.50	0.68	14.53
**M 5**	66.45	62.27	47.52	78.91	0.82	13.08	83.06	0.85	14.71
**Mitjana**	67.00	62.90	48.28	79.31	0.73	12.91	83.39	0.75	14.47
**Desv.**	0.4461	0.5269	0.7019	0.3314	0.0764	0.2199	0.2770	0.0789	0.2947

	**PHB cycle 3**								
	**R (x)**	**R (y)**	**R (z)**	**L**	**a**	**b**	**L***	**a***	**b***
**M 1**	62.53	57.88	43.03	76.08	1.66	13.66	80.67	1.74	15.68
**M 2**	63.77	59.17	44.28	76.92	1.54	13.55	81.38	1.60	15.46
**M 3**	62.68	57.79	42.25	76.02	1.78	14.32	80.62	1.86	16.52
**M 4**	63.22	58.57	43.53	76.53	1.58	13.75	81.05	1.65	15.76
**M 5**	63.74	59.44	45.37	77.10	1.37	12.77	81.53	1.43	14.47
**Mitjana**	63.19	58.57	43.69	76.53	1.59	13.61	81.05	1.66	15.58
**Desv.**	0.5778	0.7419	1.1943	0.4847	0.1516	0.5557	0.4088	0.1604	0.7370

	**PHB cycle 4**								
	**R (x)**	**R (y)**	**R (z)**	**L**	**a**	**b**	**L***	**a***	**b***
**M 1**	54.01	49.00	34.54	70.00	2.73	14.45	75.45	2.91	17.34
**M 2**	54.94	50.06	35.59	70.76	2.43	14.32	76.11	2.59	17.08
**M 3**	52.10	47.00	32.37	68.55	2.90	14.94	74.19	3.11	18.18
**M 4**	52.70	47.62	32.87	69.00	2.76	14.96	74.58	4.96	18.15
**M 5**	52.23	47.22	32.83	68.72	2.80	14.66	74.33	3.01	17.78
**Mitjana**	53.20	48.18	33.64	69.41	2.72	14.67	74.93	3.32	17.71
**Desv.**	1.2333	1.3071	1.3670	0.9426	0.1764	0.2863	0.8205	0.9395	0.4881

	**PHB cycle 5**								
	**R (x)**	**R (y)**	**R (z)**	**L**	**a**	**b**	**L***	**a***	**b***
**M 1**	48.38	43.36	29.40	65.85	3.19	14.84	71.80	3.47	18.39
**M 2**	47.80	42.75	28.82	65.38	3.30	14.91	71.38	3.59	18.55
**M 3**	48.98	43.94	29.92	66.29	3.18	14.80	72.19	3.45	18.27
**M 4**	48.26	43.32	29.59	65.82	3.15	14.60	71.77	3.43	18.06
**M 5**	47.64	42.72	29.23	65.36	3.26	14.45	71.37	3.55	17.90
**Mitjana**	48.21	43.22	29.39	65.74	3.22	14.72	71.70	3.50	18.23

Desv.	0.5285	0.5047	0.4097	0.3857	0.0619	0.1899	0.3414	0.0687	0.2585

	PHB cycle 6								
	R (x)	R (y)	R (z)	L	a	b	L*	a*	b*
M 1	47.97	42.9	28.93	65.5	3.31	14.92	71.49	3.60	18.55
M 2	48.57	43.75	30.54	66.14	3.15	13.98	72.06	3.42	17.15
M 3	47.11	42.19	28.81	64.95	3.34	14.41	71.00	3.65	17.90
M 4	48.18	43.23	29.87	65.75	3.35	14.23	71.71	3.64	17.54
M 5	49.19	44.42	31.19	66.65	3.01	13.90	72.51	3.26	16.97
Mitjana	48.20	43.30	29.87	65.80	3.23	14.29	71.75	3.51	17.62
Desv.	0.7678	0.8447	1.0245	0.6429	0.1481	0.4071	0.5713	0.1696	0.6311

Table SI4. Changes of the Optical properties of PET

	PET Virgin					
	R (x)	R (y)	R (z)	L*	a*	b*
M 1	3.62	3.74	4.29	22.51	0.12	-3.09
M 2	3.79	3.9	4.49	23.32	0.55	-3.27
M 3	3.48	3.52	4.2	22.03	1.53	-3.96
M 4	3.57	3.68	4.26	22.57	0.53	-3.36
M 5	3.76	3.85	4.56	23.17	1.05	-3.94
Mitjana	3.64	3.74	4.36	22.72	0.76	-3.52
Desv.	0.1301	0.1497	0.1560	0.5256	0.5439	0.4009

	PET cycle 1					
	R (x)	R (y)	R (z)	L*	a*	b*
M 1	4.56	4.77	5.22	26.08	-0.97	-2.21
M 2	4.53	4.74	5.38	25.99	-0.56	-3.09
M 3	4.2	4.4	4.87	24.94	-0.83	-2.47
M 4	4.04	4.24	4.79	24.45	-0.67	-2.9
M 5	4.27	4.47	5.15	25.17	-0.29	-3.4
Mitjana	4.32	4.52	5.08	25.33	-0.66	-2.81
Desv.	0.2219	0.2270	0.2463	0.6982	0.2607	0.4770

	PET cycle 2					
	R (x)	R (y)	R (z)	L*	a*	b*
M 1	6.15	5.94	4.59	29.26	-1.14	6.44
M 2	6.95	6.77	5.5	31.29	-1.13	5.46
M 3	6.5	6.23	4.84	29.98	-0.71	6.39
M 4	6.41	6.22	4.97	29.97	-1.09	5.74
M 5	7.07	6.8	5.35	31.35	-0.82	6.27
Mitjana	6.62	6.39	5.05	30.37	-0.98	6.06
Desv.	0.3843	0.3773	0.3724	0.9153	0.1992	0.4358

	PET cycle 3					
	R (x)	R (y)	R (z)	L*	a*	b*
M 1	11.85	10.83	6.95	39.29	0.22	13.22
M 2	11.62	10.58	6.61	38.86	0.21	13.73
M 3	12.36	11.23	7.15	39.97	0.55	13.5
M 4	12.57	11.53	7.66	40.46	0.32	12.43
M 5	13.04	11.86	7.66	40.99	0.72	13.3
Mitjana	12.29	11.206	7.21	39.914	0.40	13.24
Desv.	0.5675	0.5164	0.4572	0.8601	0.2235	0.4919

	PET cycle 4					
	R (x)	R (y)	R (z)	L*	a*	b*
M 1	17.86	15.90	9.77	46.84	1.91	16.21
M 2	19.17	17.13	10.84	48.42	1.97	15.71
M 3	18.41	16.36	10	47.44	1.98	16.55
M 4	18.95	16.9	10.65	48.13	2.05	15.76
M 5	19.42	17.37	10.99	47.72	1.93	15.77
Mitjana	18.76	16.73	10.45	47.71	1.97	16.00
Desv.	0.6270	0.5967	0.5359	0.6145	0.0540	0.3678

	PET cycle 5					
	R (x)	R (y)	R (z)	L*	a*	b*
M 1	22.83	20.02	11.54	51.86	2.61	19.62
M 2	23.28	20.55	12.21	52.46	2.38	18.8
M 3	23.74	20.91	12.4	52.85	2.56	18.99
M 4	24.43	21.51	12.85	53.5	2.71	18.9
M 5	23.79	20.99	12.7	52.94	2.63	18.33
Mitjana	23.61	20.80	12.34	52.72	2.58	18.93
Desv.	0.5998	0.5530	0.5124	0.6085	0.1232	0.4630

	PET cycle 6					
	R (x)	R (y)	R (z)	L*	a*	b*
M 1	24.63	21.12	11.16	53.09	3.70	22.82
M 2	25.63	22.13	12.01	54.17	3.4	22.29
M 3	25.08	21.65	11.78	53.65	3.45	22.04
M 4	24.58	21.09	11.11	53.05	3.63	22.89
M 5	25.17	21.57	11.12	53.57	3.62	23.49
Mitjana	25.02	21.51	11.44	53.51	3.56	22.71
Desv.	0.4312	0.4290	0.4272	0.4601	0.1283	0.5653

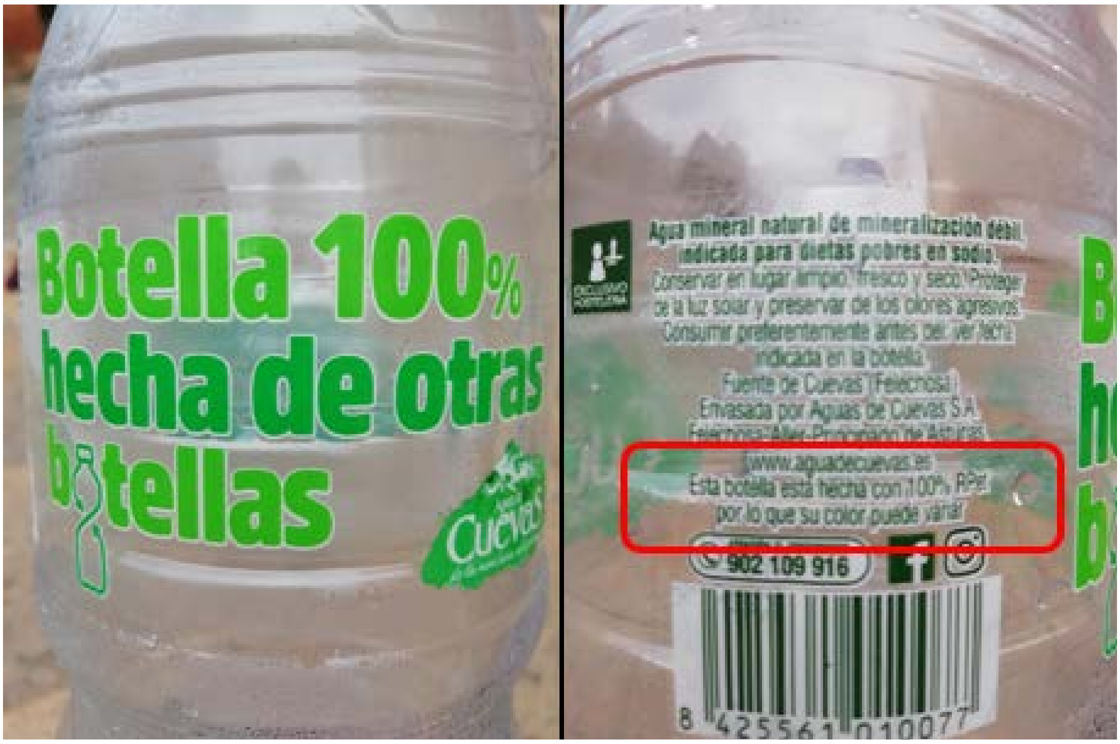

Picture SI1. Manufactured PET bottle with disregarded optical properties, clearly stating to the customers about the compromise related to the visual quality of the bottle (translation: This bottle is made from 100%RPET, therefore its color can vary)

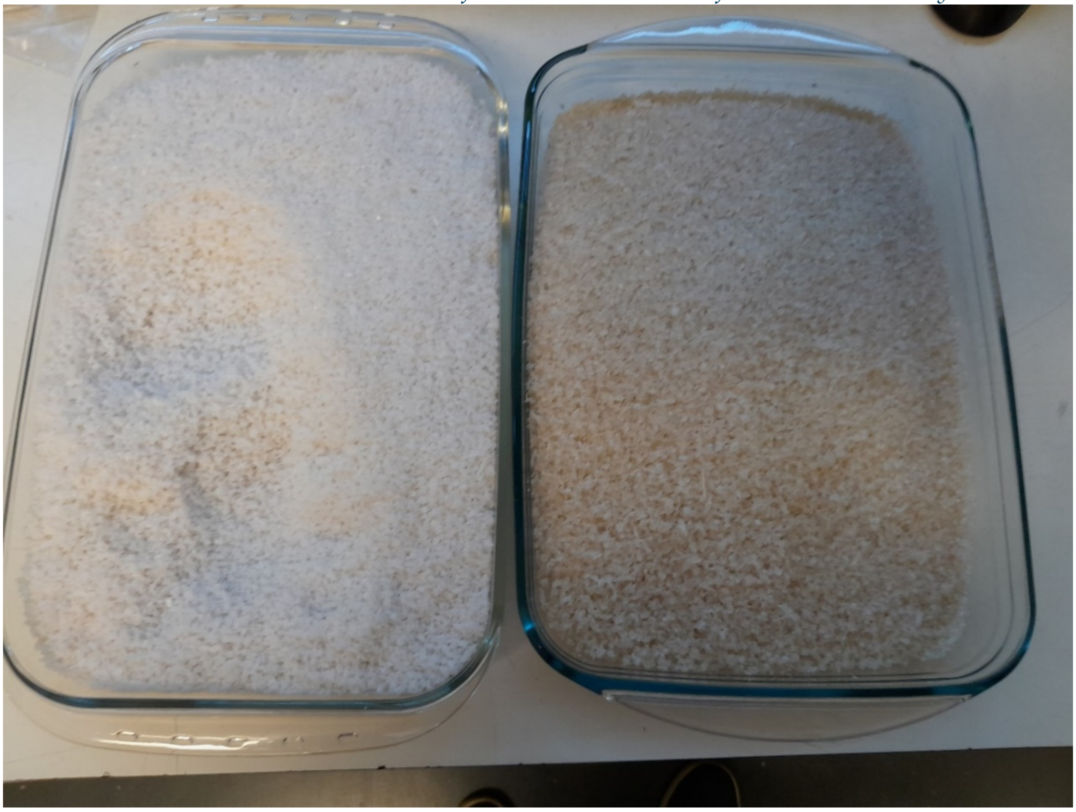

Picture SI2. Difference between the optical properties of PET between the virgin and the 1st recycling cycle
